# Altered brain dynamic in major depressive disorder: state and trait features

**DOI:** 10.1038/s41398-023-02540-0

**Published:** 2023-07-17

**Authors:** Nooshin Javaheripour, Lejla Colic, Nils Opel, Meng Li, Somayeh Maleki Balajoo, Tara Chand, Johan Van der Meer, Marina Krylova, Igor Izyurov, Tina Meller, Janik Goltermann, Nils R. Winter, Susanne Meinert, Dominik Grotegerd, Andreas Jansen, Nina Alexander, Paula Usemann, Florian Thomas-Odenthal, Ulrika Evermann, Adrian Wroblewski, Katharina Brosch, Frederike Stein, Tim Hahn, Benjamin Straube, Axel Krug, Igor Nenadić, Tilo Kircher, Ilona Croy, Udo Dannlowski, Gerd Wagner, Martin Walter

**Affiliations:** 1 Department of Psychiatry and Psychotherapy, Jena University Hospital, Philosophenweg 3, 07743 Jena, Germany; 2 Clinical Affective Neuroimaging Laboratory (CANLAB), Leipziger Str. 44, Building 65, 39120 Magdeburg, Germany; 3 German Center for Mental Health (DZPG), Jena, Germany; 4 Institute for Translational Psychiatry, University of Münster, Münster, Germany; 5 Center for Intervention and Research on adaptive and maladaptive brain Circuits underlying mental health (C-I-R-C), Jena-Magdeburg-Halle, Jena, Germany; 6 Institute of Systems Neuroscience, Heinrich Heine University Düsseldorf, Düsseldorf, 40225 Jülich, Germany; 7 Institute of Neuroscience and Medicine (INM-7), Research Centre Jülich, 52425 Jülich, Germany; 8 Department of Clinical Psychology, Friedrich Schiller University Jena, Am Steiger 3-1, 07743 Jena, Germany; 9 Department of Radiology and Nuclear Medicine, Amsterdam UMC, Amsterdam, The Netherlands; 10 Institute for Diagnostic and Interventional Radiology, Jena University Hospital, Jena, Germany; 11 Department of Psychiatry and Psychotherapy, Philipps-University Marburg, Rudolf-Bultmann-Str. 8, 35039 Marburg, Germany; 12 Center for Mind, Brain and Behavior, University of Marburg, Marburg, Germany; 13 Institute for Translational Neuroscience, University of Münster, Münster, Germany; 14 Core-Facility Brainimaging, Faculty of Medicine, University of Marburg, Marburg, Germany; 15 Department of Psychiatry and Psychotherapy, University Hospital Bonn, Venusberg-Campus 1, 53127 Bonn, Germany; 16 Department of Psychotherapie and Psychosomatic Medicine, Carl Gustav Carus University Hospital Dresden, Fetscherstr. 74, 01307 Dresden, Germany; 17 Department of Psychiatry and Psychotherapy, University of Tübingen, Tübingen, Germany; 18 Department of Psychiatry and Psychotherapy, Otto-von-Guericke University Magdeburg, Magdeburg, Germany; 19 Leibniz Institute for Neurobiology, Magdeburg, Germany; 20 Center for Behavioral Brain Sciences, Magdeburg, Germany; 21 Max Planck Institute for Biological Cybernetics, Tübingen, Germany

**Keywords:** Depression, Human behaviour

## Abstract

Temporal neural synchrony disruption can be linked to a variety of symptoms of major depressive disorder (MDD), including mood rigidity and the inability to break the cycle of negative emotion or attention biases. This might imply that altered dynamic neural synchrony may play a role in the persistence and exacerbation of MDD symptoms. Our study aimed to investigate the changes in whole-brain dynamic patterns of the brain functional connectivity and activity related to depression using the hidden Markov model (HMM) on resting-state functional magnetic resonance imaging (rs-fMRI) data. We compared the patterns of brain functional dynamics in a large sample of 314 patients with MDD (65.9% female; age (mean ± standard deviation): 35.9 ± 13.4) and 498 healthy controls (59.4% female; age: 34.0 ± 12.8). The HMM model was used to explain variations in rs-fMRI functional connectivity and averaged functional activity across the whole-brain by using a set of six unique recurring states. This study compared the proportion of time spent in each state and the average duration of visits to each state to assess stability between different groups. Compared to healthy controls, patients with MDD showed significantly higher proportional time spent and temporal stability in a state characterized by weak functional connectivity within and between all brain networks and relatively strong averaged functional activity of regions located in the somatosensory motor (SMN), salience (SN), and dorsal attention (DAN) networks. Both proportional time spent and temporal stability of this brain state was significantly associated with depression severity. Healthy controls, in contrast to the MDD group, showed proportional time spent and temporal stability in a state with relatively strong functional connectivity within and between all brain networks but weak averaged functional activity across the whole brain. These findings suggest that disrupted brain functional synchrony across time is present in MDD and associated with current depression severity.

## Introduction

Major Depressive Disorder (MDD) is a complex psychiatric condition characterized by various core symptoms, such as inflexible mood and a propensity to fixate on negative stimuli. These symptoms may encompass further symptoms or be accompanied by them, which can manifest differently in each individual. Investigating the momentary brain functional dynamics during rest, when the individual’s thoughts are not guided in tasks, can offer valuable insights into the underlying neurobiological mechanisms of MDD. Although numerous studies have contributed to our understanding of the association between depressive symptoms and the underlying neurobiological mechanisms of MDD, our knowledge in this area remained limited [1–7].

Resting-state functional magnetic resonance imaging (rs-fMRI) is one non-invasive approach to investigate the underlying changes in brain function associated with MDD [5]. Static functional connectivity (FC), a classical rs-fMRI parameter, indicates the degree of the coherent patterns of fMRI signal fluctuations by calculating correlation coefficients between the time-series of predefined brain regions [8]. Studies using this approach to study MDD have found disrupted FC within and between brain networks including the default mode (DMN), the frontoparietal (FPN), and salience (SN) networks. Altered FC within and between these functional brain networks is associated with specific depressive symptoms, such as negative affect and negative thoughts [1, 9–11]. Furthermore, whole-brain studies showed FC alteration within and between dorsal attention (DAN), somatosensory motor (SMN) and visual (VN) networks to be related to the overall depression severity [12, 13].

One core symptom of MDD is diminished fluctuation in the mood with the dominance of the negative affect state and less experiencing positive emotions [14]. This persistence of lower mood can be interlinked to rigid attention towards negative stimuli, which might also contribute to the perpetuation of negative thoughts and combined with disruption in the cognitive process prevents the patients to leave this state [15, 16]. Directing attention towards negative stimuli can also extend to bodily sensations and may intensify the perception of bodily discomfort, contributing to symptoms such as fatigue, agitation, sleep disturbances, and other somatic complaints commonly observed in individuals with MDD [17–20]. These patterns are not only relevant to the transient states of depression but also to the enduring depressive traits. Disrupted cognitive processes, distorted attention and maintained negative mood and further might be observed both as state-dependent symptoms in individuals experiencing depressive episodes and as trait-like tendencies in individuals with a predisposition to depression [21–23]. The rigidity or instability of specific dynamic patterns of functional interactions among brain regions could contribute to both the acute manifestations of depressive states and the underlying trait-related vulnerabilities [1, 4, 24, 25]. Therefore, studying the fundamental dynamics of brain function could provide additional knowledge about the mechanisms in the brain that are related to both specific depressive symptoms and the overall trait and state nature of depression [6, 26, 27].

Capturing the temporal variability of reoccurring functional activity and connectivity patterns (i.e., spatial states) is critical for understanding the dynamic organization of the brain [28]. The temporal features related to the recurring spatial states can be quantified by fractional occupancy (FO), the proportion of time spent in any of the states characterized by functional activity or connectivity, or the average lifetime of each state (ALT), a parameter of stability of the given spatial brain state and switching rate (SR) measures a ratio of overall transitioning between reoccurring functional states [29]. These dynamic patterns of specific brain connectivity known as spatiotemporal measures have already been shown to be associated with thought processing, and specific cognitive and affective states [27, 30–33]. Moreover, alterations of brain dynamics patterns have been associated with post-traumatic stress disorder, schizophrenia, and Alzheimer’s disease [34–36].

Previous dynamic functional connectivity (dFC) studies on MDD yielded inconclusive findings. This might be due to specific methodological approaches such as hypothesis-testing of selected regions of interest (ROIs) or due to variation in sample characteristics [37–39]. For example, one study calculated the standard deviation (SD) of the FC values within a predefined time-window and found decreased FC variability between the medial prefrontal cortex (MPFC) and parahippocampal gyrus in MDD, while the increased FC variability between the MPFC and insula was related to ruminative processes in MDD [40]. Another study showed that a higher probability of transitioning from a state with weak FC within DMN regions to a state with strong FC within DMN regions was correlated with depression severity [41]. The inconsistency of findings can also be seen in studies at the network level, showing either greater or lower FC variability in DMN and FPN in MDD [42–44]. However, a recent whole-brain study reported greater FC variability of DMN, SMN, and subcortical regions in the MDD [45]. It has also been shown that depressed patients have lower ALT in a state with strong connectivity between SMN and DMN [46].

Apart from the characteristics of the study sample and the selection of specific brain regions, replication of dFC findings in MDD is often challenging because of differences in the methodological approaches [39, 47–49]. Typically, the aforementioned studies used the sliding-window approach. This method calculates the dFC by correlating the time-series of the selected brain regions within an arbitrary time window [50]. Hereby, the definition of an optimal time window length is challenging - a long window increases the chance of missing specific states and a short window compromises the reliability of the FC patterns [49]. In addition, another problem with the sliding-window approach is its lack of sensitivity to transient changes in the time-series [51].

One promising alternative approach to uncovering the dynamic properties of spatial states is the hidden Markov model (HMM) [51, 52]. This probabilistic model assumes that there are discrete latent spatial states for the observed time-series, which can reoccur at any time-point, and these states can be inferred by the probability distribution over a sequence of observations [53]. Previous studies using the HMM approach in healthy populations found associations between the spatiotemporal features of brain activity and behavioral characteristics or evoked emotions while watching a movie [47, 54]. Clinical studies also showed significant differences in the brain dynamic between healthy controls and patients with schizophrenia disorder [35]. Applying the HMM to study brain dynamics in MDD has revealed that compared to healthy subjects, patients stayed longer in two brain functional states with contrasting activity patterns of DMN and SMN regions [55]. However, this study did not investigate the relation of these alterations to depression severity or specific MDD symptoms.

The main aim of the present study was to investigate the functional dynamic patterns at the whole-brain level using HMM in a large sample of 314 patients with MDD and 498 control participants and to relate these putative alterations to symptom severity and specific clusters of depressive symptoms. The inclusion of a large, heterogeneous clinical cohort enabled us to gain a better understanding of the disorder and its underlying mechanisms. Based on the findings of the previous studies, we expected that patients with MDD will have altered whole-brain dynamic patterns of functional connectivity and activity compared to the control group and that altered dynamic patterns are associated with the severity of depression. We also expected that patients with MDD will have higher stability (averaged lifetime) in certain brain states as a notion of persistence of brain states, which are also characterized by the higher functional activity of DMN regions and higher connectivity within the DMN network compared to other brain networks. We additionally hypothesized that individuals with depression will have lower overall switching rate compared to healthy individuals, indicating a reduced ability to flexibly transition between different brain states.

## Methods and material

### Demographic and sample characteristics

The data is from the Marburg-Münster Affective Disorders Cohort Study (MACS), established during a bicentric study from Marburg and Münster in Germany (data freeze by 2017). All participants gave written informed consent and received financial compensation for the study participation. The study was approved by the local ethics committee of the Medical Faculties of the Universities of Marburg (AZ:07/14) and Münster (AZ:2014-422-b-S) according to the current version of the Declaration of Helsinki.

Patients were diagnosed either with lifetime or current MDD by trained clinicians according to the Diagnostic and Statistical Manual of Mental Disorders (DSVM-IV-TR) criteria using the structured clinical interview for the DSM-IV axis I disorders (SCID-I) [56]. The symptom severity of MDD was assessed in the week of scanning by the clinician-rated 17-item Hamilton Depression Rating Scale (HAMD-17) [57], and by the self-rated Beck Depression Inventory (BDI) [58]. Healthy participants were included if they had no current or former psychiatric disorders as verified using the SCID-I interview. The initial sample included 974 participants (HC: 552, MDD: 452) within the age range of 18 to 65. Participants (both MDD patients and healthy controls) were excluded from the study (Supplementary Fig. 1 - PRISMA Flow-diagram), if they had neurological or severe medical conditions, BMI < 18.5 kg/m², current substance use, verbal IQ score less than 80 assessed by Multiple-Choice Word Test-B (MWT-B) [59], and any MRI contraindication. Furthermore, MDD patients were excluded if they had MDD with psychotic features, and had psychiatric comorbidities including cannabis and substance dependence disorder, delusional disorders, and brief psychotic disorder. Patients currently treated with antipsychotics, lithium, and anticonvulsants, as well as those with incomplete MR images were also excluded. The final sample consisted of 314 patients with MDD (female: 207 (65.9%), mean age ± standard deviation (SD): 35.9 ± 13.4) and 498 HC controls (female: 296 (59.4%), mean age ± SD: 34.0 ± 12.8; Table 1).

**Table 1:** Demographic information of the study participants.

	HC (*N* = 498)	MDD (*N* = 314)
Age		
Mean (SD)	34.0 (12.8)	35.9 (13.4)
Median [Min, Max]	29.0 [18.0, 65.0]	31.0 [18.0, 65.0]
Gender		
Female	296 (59.4%)	207 (65.9%)
Male	202 (40.6%)	107 (34.1%)
BMI		
Mean (SD)	24.5 (4.40)	25.9 (5.77)
Median [Min, Max]	23.4 [18.6, 59.5]	24.4 [18.6, 46.8]
Missing	21 (4.2%)	11 (3.5%)
Total score of HAMD-17		
Mean (SD)		8.02 (6.60)
Median [Min, Max]		7.00 [0, 28.0]
Missing		2 (0.6%)
Total score of BDI		
Mean (SD)		17.1 (11.4)
Median [Min, Max]		15.0 [0, 50.0]
Missing		12 (3.8%)
Severity based on HAMD-17		
Symptomatic		150 (47.8%)
Asymptomatic		162 (51.6%)
Missing		2 (0.6%)
Antidepressants		
Off		147 (46.8%)
On		167 (53.2%)

Patients and healthy subjects were not significantly different in terms of age and gender as indicated by Kruskal–Wallis and Chi-squared tests (*p* > 0.05).

*BDI* Beck Depression Inventory, *BMI* Body mass index, *HAMD-17* 17-item Hamilton Depression Rating Scale, *HC* healthy controls, *MDD* Major depressive disorder.

For severity analyses, we categorized patients based on the symptom severity status using the HAMD-17 scores: Asymptomatic patients (HAMD-17 = < 7, *N* = 162), and patients with clinically significant depressive symptoms as symptomatic patients (HAMD-17 > 7, *N* = 150) [60]. The symptomatic group had a mean BDI score of 24.5 (SD = 9.88), while the asymptomatic group had a mean score of 10.5 (SD = 8.29). The t-test showed a significant difference between these groups (*t*-value = −13.244, *p* < 0.001).

### Scanner information

Comparable MRI sequences and acquisition parameters were performed in both sites (Marburg: 12-channel head matrix Rx-coil; Tim Trio, Siemens, Erlangen, Germany, and Münster: 20-channel head matrix Rx-coil; Prisma, Siemens, Erlangen, Germany). In both sites, the three-dimensional magnetization prepared rapid acquisition gradient echo (MPRAGE) T1-weighted sequence was acquired with slice thickness = 1.0 mm, voxel size = 1.0 × 1.0 × 1.0 mm^3^, field of view (FOV) = 256 mm and the following parameters in Marburg: acquisition time (TA) = 4.26 min, repetition time (TR) = 1.9 s, echo time (TE) = 2.26 ms, inversion time (TI) = 900 ms, 176 slices, flip angle = 9°; and Münster: TA = 4.58 min, TR = 2.13 s, TE = 2.28 ms, TI = 900 ms, 192 slices, flip angle = 8°. T2∗-weighted echo planar imaging (EPI) sequences were used to acquire whole brain rs-fMRI images with the same parameters at both sites: TR = 2000 ms, TE = 30 ms, number of volumes = 237, flip angle = 90°, and FOV = 210 mm, matrix size = 64 × 64 and voxel size = 3.3 × 3.3 × 3.8 mm³.

### Parcellation schemes and data processing

For processing anatomical images, we followed the fsl-anat pipeline using FMRIB Software Library (FSL) version 6.0 (www.fmrib.ox.ax.uk/fsl) [61]. To address the head motion and scanner artifacts, the motion confounds (24 regressors) were removed and then FMRIB’s ICA-based X-noisefier (FIX) from the FMRIB Software Library (FSL) was used to remove further motion-related and scanner artifacts [62, 63]. The global signal regression was not used [64]. The detailed pipeline of MR preprocessing is in the Supplementary Material.

**Fig. 1:**
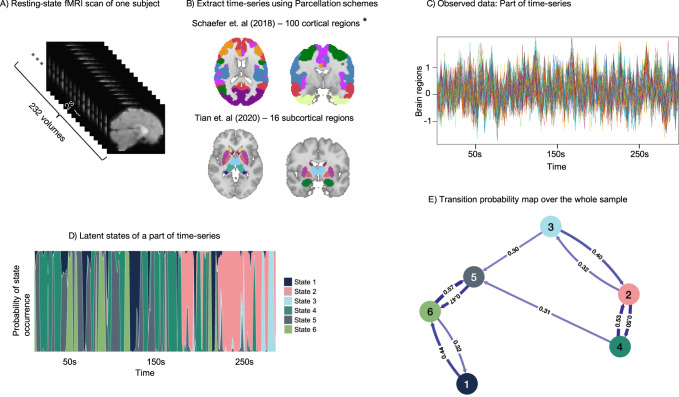
Schematic overview of the study. **A** Resting-state data from one participant representing 232 imaging volumes). **B** Schaefer et. al (2018) parcellation with 100 cortical regions and Tian et. al. (2020) parcellation with 16 subcortical regions. **C** Part of the time-series extracted from the parcellation schemes in Fig. 1B. **D** Hidden Markov Model (HMM) to calculate the probability of latent states being active at each timepoint of the observed time-series, concatenated from the whole study population. Depicted is the probability of occurrence of any state and each time-point of a part of time-series. The states do not occur sequentially and any of them might occur at any time-point. **E** Probability of transitioning from one state to any other state across groups.

To extract the time-series of the cortical regions, we used the Schaefer et al. parcellation scheme with 100 regions [65] . The 100 regions were assigned to seven brain networks according to Yeo et al. (2011) (DMN, FPN, SN, DAN, SMN, visual (VN), and limbic (LN)) [66]. Choosing this parcellation instead of an ICA data-driven approach in clinics helps with the reproducibility of other samples using the same brain parcellation. Furthermore, this specific parcellation was developed on extended resting-state data and several studies have replicated these functional networks [67]. The five regions located in the left and right orbital frontal cortex and temporal pole from the limbic network were discarded due to the signal loss in these regions. Sixteen subcortical regions based on Tian et al. (2020) were also included in our analysis. This subcortical atlas is comprised of the left and right hippocampus (HIP), amygdala (Amy), posterior and anterior thalamus (THA), nucleus accumbens (NAc), globus pallidus (GP), putamen (PUT), and caudate (CAU) [68] and the regions were assigned to a “subcortical” network. The time-series data of 812 participants with 232 time-points and 111 brain regions were combined into a 2D matrix of dimensions 188384 × 111, which was then standardized to have a mean of 0 and standard deviation of 1 for use in the HMM model (example: Fig. 1A–C).

### Hidden Markov Model

The assumption for HMM in brain dynamic analysis is that the fluctuations of time-series in brain regions can be explained by a finite number of latent states through observing the whole time-series (recurrent spatial states). Thus, each time-point can be described as a transient state. This state can either explain the next time-point or be switched to any other state. Therefore, this model can also compute the probability of being in each state at each time-point [51, 52], and provides the probability of transitioning between obtained states (Fig. 1D, E). Further, the switching rate (SR) can be computed by the rate of transitioning between all states, which represents the stability of brain dynamics per participant.

The main temporal measures driven by HMM model for downstream analyses are FO, which is defined as the proportion of time each subject spends in a specific state, and ALT, which represents the average number of time-points per visit to that state before switching to any other state, indicating the stability of a state [49].

**Fig. 2:**
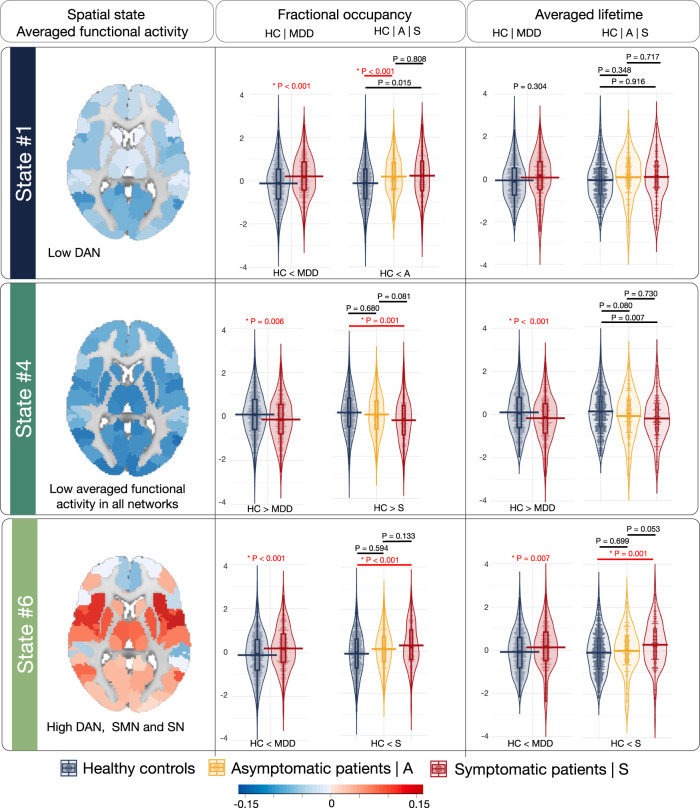
Group comparisons of the temporal features. Applying the hidden Markov model (HMM) resulted in six spatial states, with the brain map of averaged functional activity represented for each state (blue to red is indicating the negative to positive averaged functional activity, range −0.15 to 0.15). This figure contains the finding of fractional occupancy and averaged lifetime of state #1, #4 and #6 and the findings related to states #2, #3 and #5 can be found in Supplementary Fig. 4. The range of −0.15 and 0.15 for the averaged functional activity represents the level of functional activity observed during a particular state in the current dataset. In general, the magnitude and direction of the values can indicate the degree and type of neural activity occurring during a particular state. The positive values may indicate increased neural activity, while negative values may indicate decreased activity. Functional activity is averaged blood-oxygen-level-dependent (BOLD) time-series at that state for each region. The violin plots represent the group comparisons (HC vs. all MDD-diagnosed patients and HC vs. asymptomatic or symptomatic patients) of the temporal features (fractional occupancy and averaged lifetime). The value on the top of each comparison is an uncorrected p-value and the p-values that are significant also after the Bonferroni correction are indicated by red color and asterisks.

The HMM model uses the multivariate normal distribution to model the distribution of each hidden state. In this case, the time-series of each state have the parameters of the multivariate normal distribution of a mean vector and a covariance matrix. These parameters are referred to as the "averaged functional activity" of the state, as well as the "functional connectivity" of the state that is the product of the covariance matrix, which is a measure of the strength of the connections between the brain regions involved in that state [47]. The spatial states derived by HMM-MAR can be characterized by the corresponding functional connectivity matrices (Supplementary Fig. 2), which can appear at any time-point, and by averaged BOLD time-series (averaged functional activity) (Fig. 2 and Supplementary Figs. 3 and 4).

We used the Hidden Markov Model - Multivariate Autoregressive (HMM-MAR) toolbox implemented in MATLAB (https://github.com/OHBA-analysis/HMM-MAR) to perform variational Bayes inversion of HMM with 500 cycles to define states by multivariate Gaussian distribution [49], the finite number of states should be set as a prior in the model. Therefore, we ran HMM for the number of states ranging from 5 to 12 (run k = 5–12) as suggested by previous studies. For each run (k), we did at least five repetitions, i.e., computing the whole model. The final number of states was identified by assessing the summary statistics of the proportion of time visited per state for each run and repetition [47, 49, 69]. The repetitions were done with different initializations to help identify the optimal model and reduce the risk of getting stuck in a local minimum. Applying HMM with an a-priori number of more than 6 states did not demonstrate any occupation in the additional states with the minimum free energy. Therefore, we used the 6 states model for group analyses.

### Statistical analyses

For comparing the HMM indices, fractional occupancy (FO), averaged lifetime (ALT), and switching rate (SR) between patients with MDD and healthy controls, the fixed-effect linear model was used. The factor group (MDD, HC), and variables age and sex were set as fixed-effect variables. The FO, ALT of each state, and SR were set as dependent variables. Prior to statistical comparisons, the dependent variables were inverse normal transformed [70].

To better understand the dynamic characteristics in patients with different depression severity, we compared asymptomatic patients, patients with clinically significant depressive symptoms (symptomatic), and HCs to explore the differences in HMM indices based on clinician-rated depressive symptoms.

Following this, we also conducted exploratory analyses. We investigated the associations between the self-rated BDI total score and FO and ALT of spatial states, which were significant at the level of group comparisons (MDD vs. HC) using Pearson’s correlation. To explore the association between the FO and ALT of the states and the self-rated depression symptom categories, we used structured factor analyses (SFA) with the varimax rotation to find the main factors of symptoms based on the BDI. Then, the pairwise correlation between the factors (eigenvalues) and FO and ALT of the states was calculated (see Supplementary Material and Supplementary Table 1). Additionally, a literature-based two-factor solution was also tested [71] (Supplementary Table 2).

We tested the association between other clinical features, namely the number of episodes and age of onset with the FO.

Lastly, to elucidate the potential effect of antidepressant medication, we compared the FO and ALT of asymptomatic and symptomatic groups for medication status.

We used the Bonferroni correction to adjust for multiple comparisons by dividing the p-value threshold by the number of states for each temporal feature. We conducted post-hoc analyses using the Tukey method for each temporal feature and state.

The effect sizes of the main comparisons (MDD vs. HC) were computed using Cohen’s d with 2000 resamples for a 95% confidence interval [72].

To help with the interpretability of findings, we compared the average of pairwise FC within and between resting-state networks (RSN) among the six states using t-tests and adjusted the p-values for Bonferroni multiple comparisons (Supplementary Table 3 and 4).

## Results

### Spatial states and transition probability

The applied HMM model resulted in six unique spatial states characterized by specific functional connectivity (Supplementary Fig. 2 and Supplementary Tables 3 and 4) and averaged functional activity (the region-network sorted heatmap in Supplementary Fig. 3; brain maps in Fig. 2 and Supplementary Fig. 4).

State #1: FC within and between all networks is weaker compared to state #4 but relatively stronger compared to all other states. In this state, the networks have weaker within FC than the between networks connectivity compared to other states. In this state, DAN has lower averaged functional activity (AFA) compared to all other networks where the AFA is relatively zero.

State #2: The FC within all networks is relatively weaker compared to states #1 and #4 but stronger than in states #3, #5 and #6. The AFA in all networks is around zero.

State #3: In relation to their respective within FCs, the FCs of SMN, VN and DAN are relatively stronger to other networks (SN, FPN, DMN and subcortical regions) compared to all other states. The AFA in all networks is higher compared to all other states.

State #4: This can be described by higher FC within all networks and weaker connectivity between networks. However, the averaged functional activity of all brain regions in this network is lower relative to all other networks.

State #5: The FC of all networks in this state is relatively higher than state #6 but lower than all other networks. The AFA is around zero in all regions of brain networks.

State #6: The FC within all networks is weaker relative to all other states. The FC within SMN and SN and DAN are lower than the FC of these networks with all other networks compared to all other states. However, the AFA in SMN, SN and DAN is higher in this state relative to all other states.

Furthermore, transitioning between the spatial states is not sequential or random (Fig. 1E and heatmap of transition probability of the entire data: Supplementary Fig. 5). The transitions between the states were qualitatively different between healthy controls, asymptomatic, and symptomatic patients (Supplementary Fig. 6).

### Comparisons of dynamic features in MDD vs. HC

#### Fractional occupancy

Patients with MDD showed significantly higher FO in states #1 and #6 compared to controls adjusted for age and gender, Bonferroni corrected (state #1: *t*-value = 4.08, effect-size = 0.33, *p* < 0.001; and state #6: *t*-value = 3.79, effect-size = 0.30, *p* < 0.001). Control subjects exhibited higher FO in states #4 and #2 compared to MDDs (state #4: *t*-value = 2.70, effect-size = 0.23, *p* = 0.006; #2: *t*-value = 2.60, effect-size = 0.21 *p* = 0.009). However, the difference in FO of state #2 was significant only at an uncorrected level. Fractional occupancy of states #3 and #5 was not significantly different between HCs and MDDs (states #1, #4 and #6: Fig. 2; states #2, #3 and #5 Supplementary Fig. 4).

### Averaged lifetime

We also compared the averaged lifetime of the states between groups, which indicates state stability. Patients with MDD exhibited significantly higher ALT in state #6 (*t*-value = 2.66, effect-size = 0.21, *p* = 0.007) and significantly lower ALT in states #2 and #4 compared to healthy controls (state #2: *t*-value = 3.21, effect-size = 0.25, *p* = 0.001; #4: *t*-value = 3.32, effect-size = 0.27, *p* < 0.001); adjusted for age and gender, Bonferroni corrected (states #1, #4 and #6: Fig. 2; states #2, #3 and #5 Supplementary Fig. 4).

### Switching rate

The overall switching rate (SR) was not significantly different between the groups. (Supplementary Table 5: MDD vs. HC and Supplementary Table 6: HC vs. Symptomatic vs Asymptomatic)

### Comparisons of dynamic FC features in groups with different depression severity

#### Fractional occupancy

The linear models indicated significant overall group differences (symptomatic vs. asymptomatic vs. HC) regarding the FO of the three originally significantly different states (state #1: *F*-value = 13.88, *p* < 0.001; state #4: *F*-value = 8.19, *p* < 0.001; state #6: *F*-value = 10.51, *p* < 0.001; Bonferroni corrected). Pairwise contrast with Tukey’s test indicated that these differences are mainly driven by patients with current depressive symptoms (symptomatic), except for state #1, in which the asymptomatic patients showed higher FO compared to HC (state #1, asymptomatic > HC: *t*-value = 3.56, *p* < 0.001; state #4, symptomatic < HC: *t*-value = 3.40, *p* = 0.001; state #6, symptomatic > HC: *t*-value = 3.74, *p* < 0.001; Bonferroni corrected; (states #1, #4 and #6: Fig. 2; states #2, #3 and #5 Supplementary Fig. 4)).

### Averaged lifetime

The linear models with the ALT showed overall significant differences between the two states (state #4: *F*-value = 7.81, *p* < 0.001; state #6: *F*-value = 7.89, *p* < 0.001; Bonferroni corrected). The post-hoc t-tests revealed that symptomatic patients were only significantly different from healthy controls in state #6 (state #6, symptomatic > HC: *t*-value: 3.59, *p* = 0.001, Bonferroni corrected). However, comparisons between asymptomatic patients and HC or symptomatic groups showed no significant differences (states #1, #4 and #6: Fig. 2; states #2, #3 and #5 Supplementary Fig. 4)).

### Correlations between depression severity and temporal features

#### Fractional occupancy

**Fig. 3:**
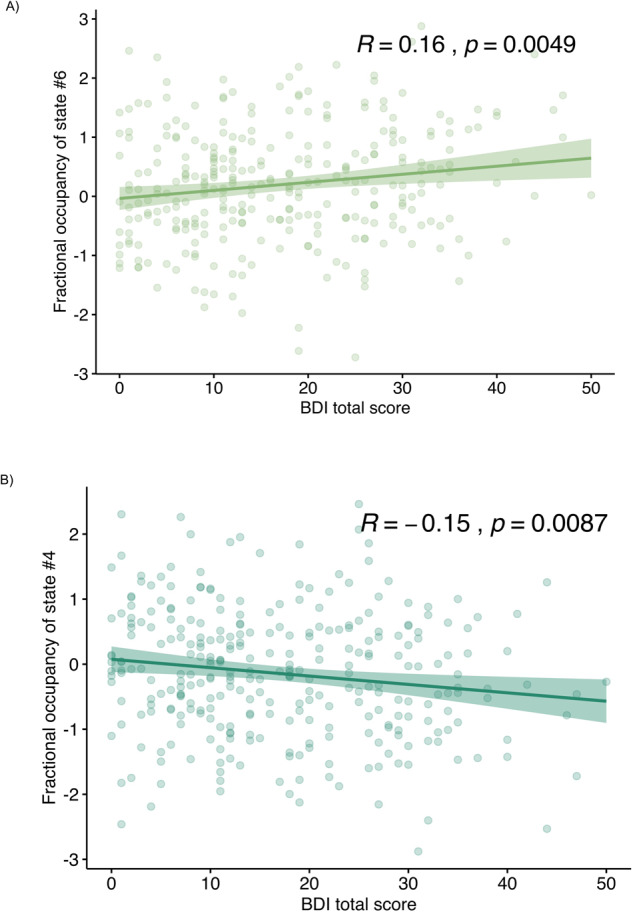
Correlation of fractional occupancy with BDI total score in MDD patients. Each scatterplot shows each state’s fractional occupancy and the BDI total scores in MDD patients. As it is indicated by the *R*-value and *p*-values on the top of each plot, **A** FO of state #6 positively correlated and **B** FO of state #4 is negatively correlated with the BDI total score. The correlations of FO of other states (#1, #2, #3, and #5) are not significant.

Depression severity, as assessed by the HAMD-17 questionnaire, was not significantly related to temporal characteristics. However, the FO of state #6 was positively correlated with the self-rated BDI total score (*R* = 0.16, *p* = 0.005; Fig. 3A). The FO of state #4 was negatively correlated with the BDI total score (*R* = −0.15, *p* = 0.008, Fig. 3B). The FO of other states were not significantly correlated with the BDI total score.

### Averaged lifetime

The correlation of ALT of state #6 and the total score of BDI (*R* = 0.16, *p* = 0.0042) was significantly positive. In contrast, the ALT of other states did not show any significant correlations (Supplementary Fig. 7).

### Switching rate

The switching rate was not associated with the BDI total score (Supplementary Fig. 8).

### Categories of depressive symptoms and temporal features

We explored the association between specific categories of depressive symptoms and FO. For that, we identified three main symptom factors in the BDI, which we labeled based on the item composition as “negative self-view”, “social and cognitive symptoms”, and “negative affect”. The pairwise Pearson correlation between Bartlett scores of the three factors and the FO and ALT of six states did not show significant correlations (respectively: Supplementary Figs. 9 and 10). None of the associations between FO and ALT of the six states and the literature-based two-factor solution [71] was significant (*p*-value < 0.05).

### Number of episodes, age of onset and fractional occupancy

For further exploratory analyses, we also compared the FO of the six states and specific MDD characteristics including categories of the number of episodes (one episode, two episodes and three or more episodes) and healthy controls (Supplementary Table 7). We found that patients with three or more episodes had higher FO in states #1 and #6 compared to HC (state #1, One episode > HC: *t*-value = 0.67, *p* = 0.001 and three or more >HC: *t*-value = 3.31, *p* = 0.005; state #6, Three or more >HC: *t*-value = 3.05, *p* = 0.001).

We also correlated the age of onset (the age of the first MDD diagnosis) and the fractional occupancy of states adjusted for age and gender in the MDD-diagnosed sample. We did not find a significant association between the FO of any state and age of onset.

### Effect of antidepressants on temporal assessments

We compared the potential effect of medication on FO and ALT within each group of symptomatic and asymptomatic (respectively: Supplementary Figs. 11 and 12). The finding indicated that patients with antidepressants did not significantly differ from unmedicated patients in FO and ALT. We also examined the FO of six states between five groups for additional investigation of antidepressant effect: medicated asymptomatic, unmedicated asymptomatic, medicated symptomatic, unmedicated symptomatic, and healthy control (Supplementary Figs. 13 and 14). In summary, the findings indicated that symptomatic and asymptomatic patients using antidepressants have higher FO and ALT in state #6. Symptomatic patients using antidepressants have a significantly lower FO and ALT compared to HC in state #4.

## Discussion

This study demonstrated that patients with major depressive disorder have different brain dynamic properties than healthy controls. By applying the hidden Markov model, six unique spatial states characterized by functional connectivity as well as averaged functional activity were obtained in the whole study sample. Comparisons of dynamic measures revealed that patients with MDD compared to healthy controls have higher fractional occupancy (FO) and averaged lifetime (ALT) in state #6, which is characterized by the weaker whole-brain functional connectome as well as higher activity of the SMN, SN, and DAN networks. Healthy subjects, in contrast, exhibited higher FO and ALT in state #4 with stronger FC within and between all networks, as well as relatively lower functional activity in all brain networks. Importantly, the alterations in MDD were largely driven by the severity of depressive symptoms. Furthermore, the significant association of self-rated depression severity in patients and temporal features of this state pointed toward the specificity of this finding for the self-experienced depressive state. It has been also shown that patients experiencing three or more depressive episodes have higher FO in states #1 and #6 compared to HC. However, the cluster of symptoms representing “negative self-view”, “negative affects” and “social and cognitive” were not significantly associated with the FO of any state. Furthermore, we did not find any significant differences in switching rate (SR) between patients and healthy controls.

The current findings are in contrast to previous dFC studies, which focused on the hypotheses of changes in FC variation or temporal alteration only in specific regions within the DMN, FPN, and SN [40–42, 44]. The current study implemented a whole-brain dynamic approach to address the etiological complexity of MDD. As depression is a heterogeneous disorder from both behavioral and biological aspects; each patient usually suffers from different combinations of symptoms. Therefore, associating predefined brain regions with the MDD diagnosis and depression severity or a single symptom of depression might contradict the phenotypes of this disorder and may lead to less replicable findings [6, 73, 74].

Previous dFC studies using the sliding-window approach reported altered dFC between brain regions located in SMN, DMN, FPN, and subcortical regions [43, 45, 46]. Furthermore, a recent whole-brain HMM study found different states and showed that patients with MDD have higher FO in two recurring functional brain states, one marked by higher activity of DMN and lower activity of SMN and subcortical regions, and the other state identified by higher activity of SMN and lower activity of DMN, and subcortical regions, while healthy controls have higher FO in a state with low activity of subcortical areas and high activity in DMN [55]. These studies and our study agree that the spatiotemporal alteration related to MDD diagnosis could not only be found in DMN but is also associated with alterations in other neural networks, including SMN. Our findings revealed that patients have higher FO and ALT in #6 with the higher functional activity of DAN, SMN, and SN, and relatively low FC not only within DMN but also within all other investigated brain networks. This finding indicates that this brain state is more stable in symptomatic patients. The current finding fits well with the findings of the recent static FC mega-analysis that showed lower FC mainly within the SMN, DAN, and SN in patients with MDD compared to healthy controls [12, 13].

The association between the temporal features (FO and ALT) of state #6 and self-rated depression severity might be explained by the possible increased salience for potential negative self-related thoughts emerging during the resting-state scan in MDD [1, 2, 10, 75]. We assume that the negative thoughts in symptomatic patients are distressing and thereby provoke emotional arousal [76–78]. Given the putative function of DAN, SMN, and SN, another speculative explanation may be that the higher activity in these networks is associated with a higher level of sympathetic arousal as a sign of stress reaction during the MR scanning [79, 80]. However, state #6 did not relate to any specific symptoms cluster of the self-rated severity. This may indicate a symptom-unspecific association and further studies measuring symptom categories with targeted questionnaires or behavioral tasks are needed to elucidate general and symptom-specific brain dynamics in MDD.

We also found that the FO of state #6 was associated with the recurrence of depression but not with the age of onset of MDD. These findings suggest that participants experiencing recurrent depressive episodes have a specific pattern of brain dynamics that may be, for example, associated with the proneness to ruminate, a feature which was previously related to dynamic connectivity and depression relapse. However, this is speculative and would require follow-up research [40, 81].

Unraveling neuroimaging markers of MDD can significantly impact monitoring remission, prediction of relapses and improving behavioral and pharmaceutical interventions [82, 83]. A recent case-control study investigating the biological and behavioral markers of MDD using structural, diffusion-tensor imaging, task, and rs-fMRI (static FC) found that the deviations of the univariate neuroimaging features of these modalities between patients with MDD and healthy controls are low and negligible [84]. However, the current dynamic features of rs-fMRI differed significantly between MDD patients and healthy controls and were sensitive to the severity of depression and the reoccurrence of depressive episodes. Previous classification studies using both static and dynamic FC features also substantiate the sensitivity of dynamic features in discerning psychiatric diagnoses [85, 86].

### Limitation

The sample was not balanced between asymptomatic and symptomatic MDD. Future studies should increase sample size and include longitudinal measurements to investigate within-person differences related to dynamic features and remission status. We limited the effect of medication other than antidepressants. However, the patients were treated with different types of antidepressants, with different doses and duration, both of which could affect the results of the current study. Although we removed the motion parameters in the preprocessing pipeline, the latent head motion may still affect the resulting brain states. The duration of the fMRI scan for each participant was relatively short. However, to address this limitation, we concatenated the time series of all participants and fit the HMM model on the resulting long format. This approach allowed us to capture a sufficient time-series to obtain temporal information and overcome the issue of short scan duration. The current findings suggest that future studies may benefit to include measures of the sympathetic nervous system and questionnaires related to self-referential processes during rs-fMRI scans to further investigate the specificity of associations between physiological and subjective states, and dynamic brain measures.

## Conclusion

In conclusion, our findings suggest that patients with MDD showed higher proportional time spent and temporal stability in spatial states characterized by relatively lower connectivity within and between entire brain functional networks and higher averaged functional activity of regions in SMN, SN, and DAN. The stability of this brain state might be associated with the inability to disengage from discomfort in the scanner or self-related negative thoughts leading to heightened arousal. The recurrence of this pattern could result in or be associated with the maintenance of negative mood states in patients with higher severity of depressive symptoms and multiple recurrent episodes. While the altered temporal dynamics may contribute to the persistence of negative affect and attention biases in MDD, it is noteworthy that asymptomatic individuals share similarities with healthy individuals in terms of temporal dynamics. This suggests that affective domains, such as mood and emotional processing, may show the symptomatic resemblance between asymptomatic individuals and healthy individuals, while the brain dynamics changes may appear less prominent. However, it is important to acknowledge that these interpretations are partially speculative due to the limitations of the study, including the absence of subjective assessments of experiences in the scanner and physiological measures. Future research incorporating subjective reports and physiological data could provide further insights into the relationship between temporal dynamics, attention biases, and the interplay between trait and state characteristics of MDD.

## Supplementary information

Supplemental Material
