# Supplementary material for: Altered brain dynamic in major depressive disorder: state and trait features
Source: Transl Psychiatry. 2023 Jul 17;13:261. doi: 10.1038/s41398-023-02540-0 (PMC10352359; doi:10.1038/s41398-023-02540-0)
Supplement: Supplementary file 1 — Supplemental Material [file 41398_2023_2540_MOESM1_ESM.pdf]

**Title:**

Altered brain dynamic in major depressive disorder: State and trait features

**Running title:**

Altered brain dynamic in major depressive disorder: State and trait features

**Authors:**

Nooshin Javaheripour [1], Lejla Colic [1,2], Nils Opel [1,2,3,4], Meng Li [1,4,5], Somayeh Maleki Balajoo [6,7], Tara Chand [1,5,8], Johan Van der Meer [9], Marina Krylova [1,10], Igor Izyurov [1], Tina Meller [11,12], Janik Goltermann [3], Nils R. Winter [3], Susanne Meinert [3], Dominik Grotegerd [3], Andreas Jansen [11,13], Nina Alexander [11,13], Paula Usemann [11], Florian Thomas-Odenthal [11], Ulrika Evermann [11], Adrian Wroblewski [11], Katharina Brosch [11], Frederike Stein [11,12], Tim Hahn [3], Benjamin Straube [11,12], Axel Krug [14], Igor Nenadić [11], Tilo Kircher [11], Ilona Croy [4,15], Udo Dannlowski [3], Gerd Wagner ^* [1, 4], Martin Walter ^* [1,2,4,5,16,17,18,19, 20]

1 Department of Psychiatry and Psychotherapy, Jena University Hospital, Philosophenweg 3, 07743, Jena, Germany

2 German Center for Mental Health (DZPG), Halle-Jena-Magdeburg site, Germany

3 Institute for Translational Psychiatry, University of Münster, Münster, Germany

4 Center for Intervention and Research on adaptive and maladaptive brain Circuits underlying mental health (C-I-R-C), Jena-Magdeburg-Hale, Germany

5 Clinical Affective Neuroimaging Laboratory (CANLAB), Leipziger Str. 44, Building 65, 39120, Magdeburg, Germany

6 Institute of Systems Neuroscience, Heinrich Heine University Düsseldorf, 40225 Düsseldorf, Germany

7 Institute of Neuroscience and Medicine (INM-7), Research Centre Jülich, 52425 Jülich, Germany

8 Department of Clinical Psychology, Friedrich Schiller University Jena, Am Steiger 3-1, 07743 Jena, Germany

9 Department of Radiology and Nuclear Medicine, Amsterdam UMC, Amsterdam, The Netherlands

10 Institute for Diagnostic and Interventional Radiology, Jena University Hospital, Jena, Germany

11 Department of Psychiatry and Psychotherapy, University of Marburg, Rudolf-Bultmann-Str. 8, 35039 Marburg, Germany

12 Center for Mind, Brain and Behavior, University of Marburg, Marburg, Germany

13 Core-Facility Brainimaging, Faculty of Medicine, University of Marburg

14 Department of Psychiatry and Psychotherapy, University Hospital Bonn, Venusberg-Campus 1, 53127 Bonn, Germany

15 Department of Psychotherapie and Psychosomatic Medicine, Carl Gustav Carus University Hospital Dresden, Fetscherstr. 74, 01307 Dresden, Germany

16 Department of Psychiatry and Psychotherapy, University of Tübingen, Tübingen, Germany

17 Department of Psychiatry and Psychotherapy, Otto-von-Guericke University Magdeburg, Magdeburg, Germany

18 Leibniz Institute for Neurobiology, Magdeburg, Germany

19 Center for Behavioral Brain Sciences, Magdeburg, Germany

20 Max Planck Institute for Biological Cybernetics, Tübingen, Germany

^ These authors contributed equally: Gerd Wagner, Martin Walter

* Corresponding authors:

1- Gerd Wagner: wagner.gerd@uni-jena.de

2- Martin Walter: Martin.Walter@med.uni-jena.de

# Methods

## Neuroimaging data processing

For processing anatomical images, we followed the fsl-anat pipeline using the FMRIB Software Library (FSL) version 6.0 (www.fmrib.ox.ax.uk/fsl) [1]. In the first step, we reoriented the anatomical images to be comparable with the standard template (MNI152) and removed the neck and lower head. Whilst, we corrected images for spatial intensity variation including bias field and radio frequency inhomogeneity. For registering images in standard space (MNI152), the FMRIB's nonlinear image registration tool (FNIRT) with 12 degrees of freedom and a warp resolution of 10 mm was applied. Before tissue segmentation (CSF, GM, and WM), the non-brain tissues were extracted.

For preprocessing resting-state functional images, the high-pass filter of 0.01 Hz and for spatial smoothing a Gaussian kernel of full-width-at-half-maximum (FWHM) of 5 mm was applied and the first five volumes were discarded. Then, functional images were registered to the corresponding anatomical processed images using FMRIB's Linear Image Registration Tool (FLIRT) [2,3] and subsequently to MNI152 standard space with FMRIB's Nonlinear Image Registration Tool (FNIRT). Then, spatial independent components (IC) associated with the time course of rs-fMRI images were obtained with automatic estimation of the number of components using Multivariate Exploratory Linear Optimized Decomposition into Independent Components (MELODIC) at the subject level. To remove artifactual components, FMRIB's ICA-based X-noisefier (FIX) from the FMRIB Software Library (FSL) was used [4,5]. We did not use global signal regression.

### Transition probability maps

The transitions are non-random and moving between specific states is easier than between others. Therefore, we applied a threshold (probability > 0.2) to show only the smoother trajectories. The hidden Markov model was applied to the whole population regardless of the diagnosis (both MDD patients and healthy controls). This approach facilitates comparing the temporal features in common spatial states. Here, other than calculating the transition probability over the population, we also subset the data based to observe if different groups have different transition paths. Since the HMM model is at the population level, applying the statistical test to compare the transition probability maps is not possible (Supplementary Figure 5 and heatmap of transition probability of the entire data: Supplementary Figure 9).

### Symptom clustering

For exploring the association of the fractional occupancy (FO) of the states and the self-rated depression cluster of symptoms, we used structured factor analyses with the varimax rotation to find the main factors of symptoms based on the Beck depression inventory II (BDI). Then, the pairwise Pearson's correlation between the factors (eigenvalues) and fractional occupancy (FO) and average life time (ALT) of the states was calculated.

## Results

### Transition probability in healthy, asymptomatic and symptomatic groups

Qualitatively observing the results regarding the transition probability indicated that a subset of patients with clinically relevant levels of depressive symptoms has a slightly

different trajectory of moving between states than asymptomatic patients and healthy groups. We observed that transitioning towards states #1 and then #6 (FO of states #1 and #6: MDD > HC) is more probable for symptomatic patients than for asymptomatic patients and healthy controls. Whereas, it is more probable for healthy participants to move from state #1 to state #5 compared to both asymptomatic and symptomatic patients. However, this trajectory is less probable for asymptomatic patients. This observation highlights that asymptomatic patients might not have similar brain dynamic patterns to healthy controls or symptomatic patients.

**Symptom clusters and FO and ALT**

We identified three main factors (loading > 0.5). These factors included Negative self-view (body image change, sense of failure, guilt, self-dislike, self-accusation); Social and cognitive symptoms (indecisiveness, work difficulty, fatigability, self-dissatisfaction), and Negative affect (sadness, crying, pessimism). None of the factors showed a significant association with FO and ALT ($p > 0.05$), and the R-values ranged between -0.2 and 0.2 (Supplementary Figures 9 and 10).

**Reference:**

1. Jenkinson M, Beckmann CF, Behrens TEJ, Woolrich MW, Smith SM. FSL. NeuroImage. 2012;62:782–790.
2. Jenkinson M, Smith S. A global optimisation method for robust affine registration of brain images. Med Image Anal. 2001;5:143–156.
3. Jenkinson M, Bannister P, Brady M, Smith S. Improved Optimization for the Robust and Accurate Linear Registration and Motion Correction of Brain Images. NeuroImage. 2002;17:825–841.
4. Salimi-Khorshidi G, Douaud G, Beckmann CF, Glasser MF, Griffanti L, Smith SM. Automatic Denoising of Functional MRI Data: Combining Independent Component Analysis and Hierarchical Fusion of Classifiers. NeuroImage. 2014;90:449–468.
5. Griffanti L, Salimi-Khorshidi G, Beckmann CF, Auerbach EJ, Douaud G, Sexton CE, et al. ICA-based artefact removal and accelerated fMRI acquisition for improved resting state network imaging. NeuroImage. 2014;95:232–247.

**Supplementary Table 1 Loading of the BDI items in each factor**

| Label | Item | Factor 1 | Factor 2 | Factor 3 |
|---|---|---|---|---|
| sadness | BDI_01 | 0.283 | 0.324 | 0.684 |
| crying | BDI_10 | 0.281 | 0.337 | 0.445 |
| pessimism | BDI_02 | 0.454 | 0.269 | 0.648 |
| sense of failure | BDI_03 | 0.64 | 0.217 | 0.31 |
| self-dissatisfaction | BDI_04 | 0.402 | 0.507 | 0.412 |
| guilt | BDI_05 | 0.568 | 0.266 | 0.228 |
| punishment | BDI_06 | 0.438 | 0.235 | 0.186 |
| self-dislike | BDI_07 | 0.65 | 0.186 | 0.347 |
| self-accusations | BDI_08 | 0.77 | 0.268 | -0.065 |
| suicidal ideas | BDI_09 | 0.32 | 0.169 | 0.435 |
| irritability | BDI_11 | 0.32 | 0.391 | 0.354 |
| social withdrawal | BDI_12 | 0.425 | 0.403 | 0.368 |
| indecisiveness | BDI_13 | 0.404 | 0.593 | 0.341 |
| body image change | BDI_14 | 0.507 | 0.163 | 0.262 |
| work difficulty | BDI_15 | 0.329 | 0.696 | 0.295 |
| insomnia | BDI_16 | 0.233 | 0.347 | 0.16 |
| fatigability | BDI_17 | 0.28 | 0.635 | 0.304 |
| loss of appetite | BDI_18 | 0.102 | 0.275 | 0.315 |
| weight loss | BDI_19 | -0.032 | 0.154 | 0.17 |
| somatic preoccupation | BDI_20 | 0.252 | 0.26 | 0.104 |
| loss of libido | BDI_21 | 0.291 | 0.365 | 0.18 |

**Supplementary Table 2: labels of items based on two factors of somatic and cognitive-affective of BDI**

| BDI_II | | BDI | Factor |
|---|---|---|---|
| BDI 1 | Sadness | Sadness | Somatic |
| BDI 2 | Pessimism | Pessimism | Somatic |
| BDI 3 | Past failure | Sense of failure | Somatic |
| BDI 4 | Loss pleasure | Self-dissatisfaction | Cognitive_Affective |
| BDI 5 | Guilt feelings | Guilt | Somatic |
| BDI 6 | Punishment | Punishment | Somatic |
| BDI 7 | Self-dislike | Self-dislike | Somatic |
| BDI 8 | Self-critical | Self-accusations | Somatic |
| BDI 9 | Suicidal | Suicidal ideas | Somatic |
| BDI 10 | Crying | Crying2 | Cognitive_Affective |
| BDI 11 | Agitation | Irritability | Cognitive_Affective |
| BD1 12 | Loss interest | Social withdrawal | Cognitive_Affective |
| BDI 13 | Indecisive | Indecisiveness | Cognitive_Affective |
| BDI 14 | Worthless | Body image change | Somatic |
| BDI 15 | Loss energy | Work difficulty | Cognitive_Affective |
| BDI 16 | Sleep | Insomnia | Cognitive_Affective |
| BDI 17 | Irritable | Fatigability | Cognitive_Affective |
| BDI 18 | Appetite | Loss of appetite | Cognitive_Affective |
| BDI 19 | Concentration | Weight loss | Cognitive_Affective |
| BDI 20 | Tiredness | Somatic preoccupation | Cognitive_Affective |
| BDI 21 | Sex interest | Loss of libido | Cognitive_Affective |

**Supplementary Table 3: T-values of the t-tests comparing the averaged functional connectivity of within networks between each two states**

| Within Network | S_1 vs S_2 | S_1 vs S_3 | S_1 vs S_4 | S_1 vs S_5 | S_1 vs S_6 | S_2 vs S_3 | S_2 vs S_4 | S_2 vs S_5 | S_2 vs S_6 | S_3 vs S_4 | S_3 vs S_5 | S_3 vs S_6 | S_4 vs S_5 | S_4 vs S_6 | S_5 vs S_6 |
|---|---|---|---|---|---|---|---|---|---|---|---|---|---|---|---|
| DAN | -0.94 ns | 1.52 ns | -5.11 **** | 5.68 **** | 7.01 **** | 2.47 ns | -4.12 ** | 6.56 **** | 7.84 **** | -6.75 **** | 4.3 ** | 5.73 **** | 10.81 **** | 11.93 **** | 1.61 ns |
| DMN | 6.96 **** | 13.28 **** | -2.65 ns | 9.97 **** | 8.81 **** | 5.88 **** | -9.07 **** | 2.14 ns | 1.62 ns | -15.05 **** | -4.26 ** | -4.35 ** | 12.03 **** | 10.85 **** | -0.41 ns |
| FPN | 2.33 ns | 4.60 *** | -0.38 ns | 3.01 ns | 3.09 ns | 2.47 ns | -2.76 ns | 0.69 ns | 1.04 ns | -5.07 *** | -1.84 ns | -1.2 ns | 3.47 ns | 3.5 ns | 0.44 ns |
| SMN | -0.26 ns | 1.15 ns | -2.45 ns | 3.87 * | 4.32 ** | 1.46 ns | -2.27 ns | 4.25 ** | 4.69 *** | -3.63 * | 2.84 ns | 3.35 ns | 6.18 **** | 6.51 **** | 0.64 ns |
| SN | 2.43 ns | 5.04 *** | -2.7 ns | 3.67 * | 5.26 **** | 2.54 ns | -5.21 **** | 1.11 ns | 2.71 ns | -8.03 **** | -1.55 ns | 0.13 ns | 6.68 **** | 8.3 **** | 1.72 ns |
| Sub | 2.46 ns | 4.51 ** | -3.55 * | 1.84 ns | 2.5 ns | 1.91 ns | -5.8 **** | -0.61 ns | 0.01 ns | -7.88 **** | -2.54 ns | -1.92 ns | 5.22 **** | 5.88 **** | 0.62 ns |
| VN | -1.03 ns | 1.28 ns | -7.59 **** | 4.38 ** | 8.44 **** | 2.18 ns | -6.67 **** | 5.47 **** | 9.5 **** | -7.92 **** | 2.43 ns | 6.03 **** | 11.86 **** | 15.36 **** | 4.41 ** |

*We compared the within connectivity of networks (in the left column) in every two states using the t-test and we used Bonferroni approach for multiple comparisons for all comparisons (ns= not significant, * = 0.05, ** = 0.001, *** = 0.0001, **** = 0.00001)*

**Supplementary Table 4: T-values of the t-tests comparing the averaged FC of between networks between each two states**

| Networks | S_1 vs S_2 | S_1 vs S_3 | S_1 vs S_4 | S_1 vs S_5 | S_1 vs S_6 | S_2 vs S_3 | S_2 vs S_4 | S_2 vs S_5 | S_2 vs S_6 | S_3 vs S_4 | S_3 vs S_5 | S_3 vs S_6 | S_4 vs S_5 | S_4 vs S_6 | S_5 vs S_6 |
|---|---|---|---|---|---|---|---|---|---|---|---|---|---|---|---|
| DAN-DMN | 7.68 **** | 16.64 **** | -3.59 ns | 14.04 **** | 14.77 **** | 7.58 **** | -10.32 **** | 4.3 ** | 5.42 **** | -18.35 **** | -4.52 ** | -2.79 ns | 16.06 **** | 16.69 **** | 1.67 ns |
| DAN-FPN | 8.48 **** | 11.26 **** | -0.35 ns | 7.87 **** | 8.76 **** | 2.47 ns | -8.15 **** | -1.19 ns | 0.41 ns | -10.66 **** | -3.89 * | -2.01 ns | 7.54 **** | 8.42 **** | 1.6 ns |
| DAN-SMN | -0.65 ns | 5.85 **** | -9.1 **** | 13.28 **** | 12.09 **** | 6.73 **** | -8.76 **** | 14.41 **** | 13.06 **** | -15.15 **** | 7.68 **** | 6.9 **** | 22.45 **** | 20.56 **** | -0.15 ns |
| DAN-SN | 4.99 *** | 7.41 **** | -2.5 ns | 9.39 **** | 11.22 **** | 1.87 ns | -7.2 **** | 3.87 * | 6.04 **** | -9.63 **** | 2.31 ns | 4.86 *** | 11.46 **** | 13.1 **** | 2.73 ns |
| DAN-Sub | 13.74 **** | 18.09 **** | -0.12 ns | 13.76 **** | 14.37 **** | 3.77 ns | -11.64 **** | -0.19 ns | 1.53 ns | -15.1 **** | -4.03 * | -1.91 ns | 11.61 **** | 12.4 **** | 1.73 ns |
| DAN-VN | -2.31 ns | 5.89 **** | -10.28 **** | 13.3 **** | 17.28 **** | 8.26 **** | -8.31 **** | 16.14 **** | 19.9 **** | -15.53 **** | 6.49 **** | 11.05 **** | 23.1 **** | 26.09 **** | 5.84 **** |
| DMN-FPN | 8.85 **** | 17.06 **** | -2.39 ns | 12.73 **** | 10.28 **** | 8.26 **** | -11.01 **** | 3.09 ns | 1.97 ns | -18.98 **** | -5.93 **** | -5.8 **** | 14.91 **** | 12.29 **** | -0.75 ns |

| | | | | | | | | | | | | | | | |
|---|---|---|---|---|---|---|---|---|---|---|---|---|---|---|---|
| DMN-SMN | 7.61 **** | 17.55 **** | -0.2 ns | 21.59 **** | 17.19 **** | 7.03 **** | -6.12 **** | 7.06 **** | 4.95 *** | -13.21 **** | -1.31 ns | -3.24 ns | 14.06 **** | 11.84 **** | -2.63 ns |
| DMN-SN | 10.53 **** | 20.06 **** | -5.8 **** | 16.82 **** | 15.86 **** | 9.1 **** | -15.74 **** | 4.49 ** | 4.91 *** | -24.92 **** | -5.96 **** | -4.36 ** | 22.31 **** | 20.95 **** | 1.05 ns |
| DMN-Sub | 14.89 **** | 28.81 **** | -8.23 **** | 20.29 **** | 21.29 **** | 13.45 **** | -22.76 **** | 4.02 * | 6.85 **** | -36.57 **** | -10.58 **** | -5.98 **** | 28.67 **** | 28.81 **** | 3.54 ns |
| DMN-VN | 8.49 **** | 17.07 **** | -0.23 ns | 9.14 **** | 18.25 **** | 5.95 **** | -6.53 **** | -2.74 ns | 5.36 **** | -12.2 **** | -11.39 **** | -1.29 ns | 5.62 **** | 12.06 **** | 11.97 **** |
| FPN-SMN | 13.3 **** | 18.26 **** | 3.45 ns | 15.04 **** | 11.01 **** | 4.64 ** | -8.73 **** | -0.16 ns | -2.04 ns | -13.2 **** | -5.37 **** | -6.65 **** | 9.56 **** | 6.71 **** | -2.13 ns |
| FPN-SN | 5.86 **** | 10.63 **** | -2.59 ns | 7.25 **** | 7.05 **** | 5.01 *** | -8.5 **** | 1.47 ns | 1.54 ns | -13.23 **** | -3.52 ns | -3.18 ns | 9.88 **** | 9.57 **** | 0.15 ns |
| FPN-Sub | 9.75 **** | 17.41 **** | -5.32 **** | 10.39 **** | 11.57 **** | 7.93 **** | -14.29 **** | 0.73 ns | 2.29 ns | -21.26 **** | -7.15 **** | -5.32 **** | 14.85 **** | 15.82 **** | 1.58 ns |
| FPN-VN | 12.21 **** | 15.34 **** | 2.57 ns | 4.94 *** | 11.5 **** | 2.61 ns | -7.34 **** | -8.9 **** | -0.65 ns | -9.71 **** | -12.25 **** | -3.28 ns | 0.89 ns | 6.77 **** | 8.13 **** |
| SMN-SN | 3.77 ns | 7.87 **** | -1.2 ns | 8.99 **** | 9.66 **** | 3.19 ns | -4.42 ** | 3.82 ns | 4.68 ** | -7.94 **** | 0.51 ns | 1.64 ns | 8.81 **** | 9.46 **** | 1.24 ns |
| SMN-Sub | 11.73 **** | 14.33 **** | 3.65 ns | 7.91 **** | 9.59 **** | 2.32 ns | -6.44 **** | -3.7 ns | -0.87 ns | -8.52 **** | -6.08 **** | -2.93 ns | 3.22 ns | 5.13 *** | 2.43 ns |

| | | | | | | | | | | | | | | | |
|---|---|---|---|---|---|---|---|---|---|---|---|---|---|---|---|
| SMN-VN | 2.6 ns | 3.86 * | -16.58 **** | 12.16 **** | 15.84 **** | 1.52 ns | -22.62 **** | 11.23 **** | 15.17 **** | -23.69 **** | 9.62 **** | 13.93 **** | 32.32 **** | 31.25 **** | 6.39 **** |
| SN-Sub | 8.49 **** | 15.4 **** | -4.95 *** | 9.06 **** | 10.7 **** | 6.84 **** | -13.44 **** | 0.37 ns | 2.66 ns | -20.41 **** | -6.65 **** | -3.75 ns | 14.12 **** | 15.43 **** | 2.37 ns |
| SN-VN | 7.78 **** | 9.54 **** | -1.34 ns | 6.1 **** | 9.64 **** | 0.85 ns | -7.58 **** | -3.42 ns | 1.41 ns | -8.82 **** | -4.96 *** | 0.66 ns | 5.93 **** | 9.03 **** | 5.34 **** |
| Sub-VN | 15.27 **** | 15.16 **** | 6.9 **** | 8.01 **** | 12.49 **** | -0.76 ns | -5.65 **** | -8.53 **** | -3.65 ns | -5.16 *** | -8.1 **** | -3 ns | -1.05 ns | 2.81 ns | 5.11 *** |

*We compared the between connectivity of pair of networks (in the left column) in every two states using the t-test and we used Bonferroni approach for multiple comparisons for all comparisons (ns= not significant, * = 0.05, ** = 0.001, *** = 0.0001, **** = 0.00001)*

**Table 5: Switching rate (MDD vs. HC, corrected for age and gender)**

| contrast | estimate | SE | t-value | p-value |
|---|---|---|---|---|
| HC - MDD | -0.0749 | 0.0721 | -1.0377 | 0.299 |

**Table 6: Switching rate (HC vs. Symptomatic vs Asymptomatic, corrected for age and gender)**

| contrast | estimate | SE | t-value | p-value |
|---|---|---|---|---|
| Symptomatic vs. HC | -0.04 | 0.5 | -0.8 | 0.7 |
| Symptomatic vs. Asymptomatic | -0.01 | 0.1 | -1.0 | 0.6 |
| HC vs. Asymptomatic | 0.30 | 0.4 | 0.6 | 0.8 |

**Supplementary Table 7: Comparison of MDD grouped by number of episodes and healthy controls for fractional occupancy of states**

| State | group1 | group 2 | Estimate | SE | t-value | p-value |
|---|---|---|---|---|---|---|
| state_1 | HC | One episode | -0.317 | 0.086 | -3.677 | 0.001 |
| state_1 | HC | Three or more episodes | -0.287 | 0.087 | -3.311 | 0.005 |
| state_2 | HC | Three or more episodes | 0.26 | 0.099 | 2.621 | 0.044 |
| state_4 | HC | Three or more episodes | 0.302 | 0.099 | 3.05 | 0.013 |
| state_6 | HC | Three or more episodes | -0.36 | 0.098 | -3.681 | 0.001 |

We compared the FO of six states between participants with one episode, two episodes and three or more episodes of MDD and HC adjusted for age and gender using a linear model.

**Supplementary Table 8: Comparisons of FO between MDD and HC groups adjusted for age, gender and binarized site**

| Contrast | States | Estimate | t-value | p-value |
|---|---|---|---|---|
| MDD > HC | State_1 | 0.269 | 0.061 | 0.000 |
| MDD > HC | State_2 | -0.202 | 0.07 | 0.004 |
| / | State_3 | -0.077 | 0.07 | 0.270 |
| MDD < HC | State_4 | -0.193 | 0.069 | 0.005 |
| / | State_5 | 0.026 | 0.066 | 0.688 |
| MDD > HC | State_6 | 0.271 | 0.069 | 0.000 |

Supplementary Table 6 shows the differences in fractional occupancy of different states between MDD and HC groups while controlling for age, gender, and site using the linear model. Adding site (binary of site 1 and site 2) as a covariate did not substantially change the results.

## Supplementary Figure 1: PRISMA flow-diagram for exclusion criteria

| | | |
|---|---|---|
| All Participants | Two sites including in total 974 participants:<br>MDD: 452 (female:286 (63.3%), Age (mean ± SD: 36.4 ± 13.4)) & HC: 522 (female: 313 (60.0%), Age (mean ± SD: 33.9 ± 12.7))<br>Marburg (MDD: 269, HC: 301)<br>Münster (MDD: 183, HC: 221) | |
| Technical level | No. Participants after excluding for neuroimaging files failures:<br>Total: 972<br>MDD: 451 & HC 521 | - One MDD participant: registration failure of functional file<br>- One HC participant: corrupted file |
| Clinical level | No. Participants after excluding for interview reasons:<br>Total: 812<br>MDD: 314 & HC 498 | - BMI < 18.5: 20 MDD and 15 HC<br>- More than 4 weeks between diagnosis interview and - MRI acquisition: Two HC and one MDD<br>- Unclear diagnosis: three MDD and five HC<br>- Patients with psychotic features: 11 MDD<br>- Comorbidity disorders: cannabis and poly-substance dependence disorder, delusional disorders, brief psychotic disorder: four MDD<br>- Antipsychotic: 0, Lithium: five MDD, Anti-convulsive: 9, Neuroleptic: 80 MDD and one HC |
| Included | In total 812 participants (MDD: 314 (female: 207 (65.9%), age (mean ± SD: 35.9 ± 13.4)&<br>HC: 498 (female: 296 (59.4%), age (mean ± SD: 34.0 ± 12.8)) | |

This flow diagram illustrates the exclusion and inclusion criteria of the present study and the number of subjects excluded from the study. In the end, we included 314 MDD patients and 498 healthy controls.

## Supplementary Figure 2: Functional connectivity matrices of the six data-driven states

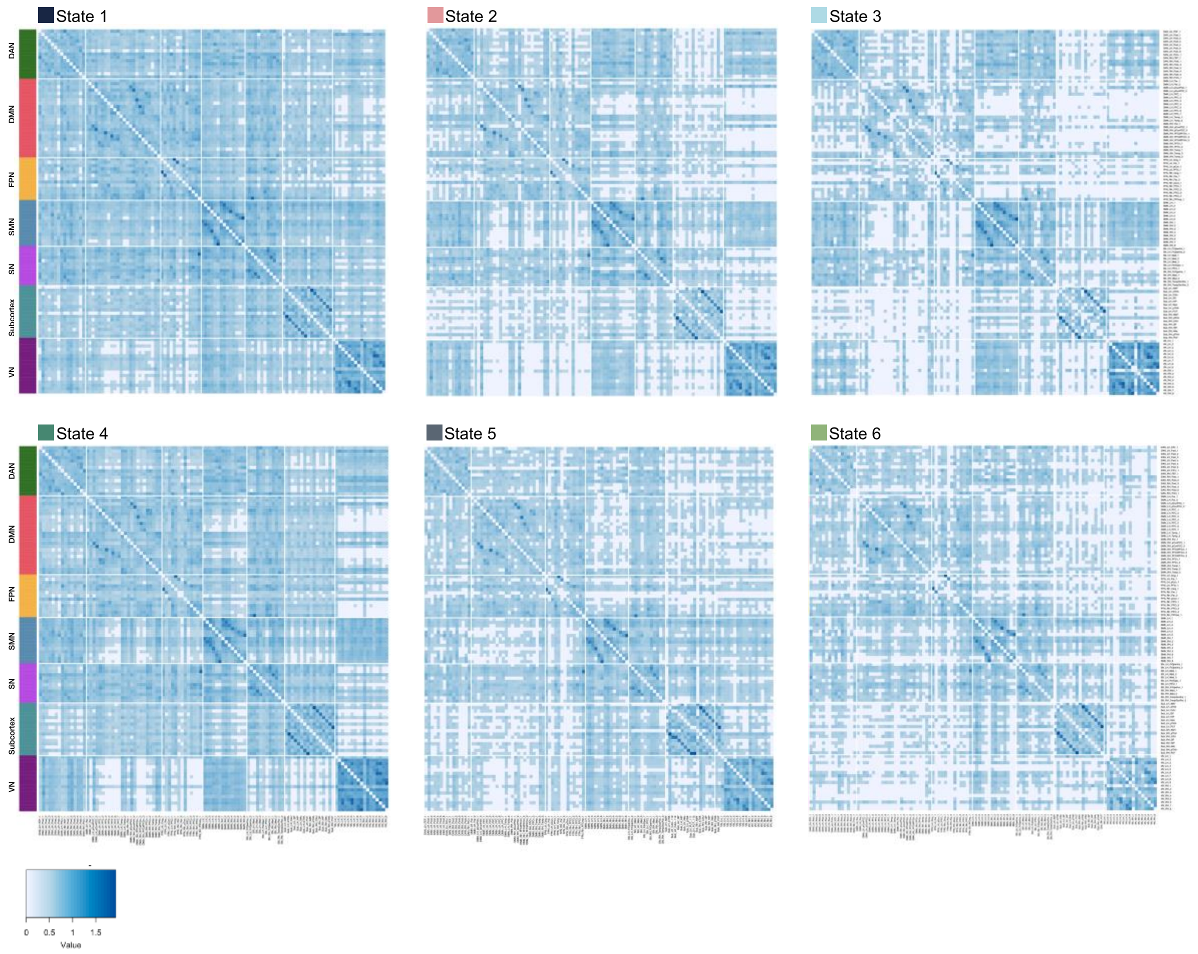


The functional connectomes of six unique spatial states are presented. To summarize the differences between the states, we compared within and between functional connectivity of the networks in Supplementary Tables 1A and 1B.

## Supplementary Figure 3: Overview of averaged functional activity of the states

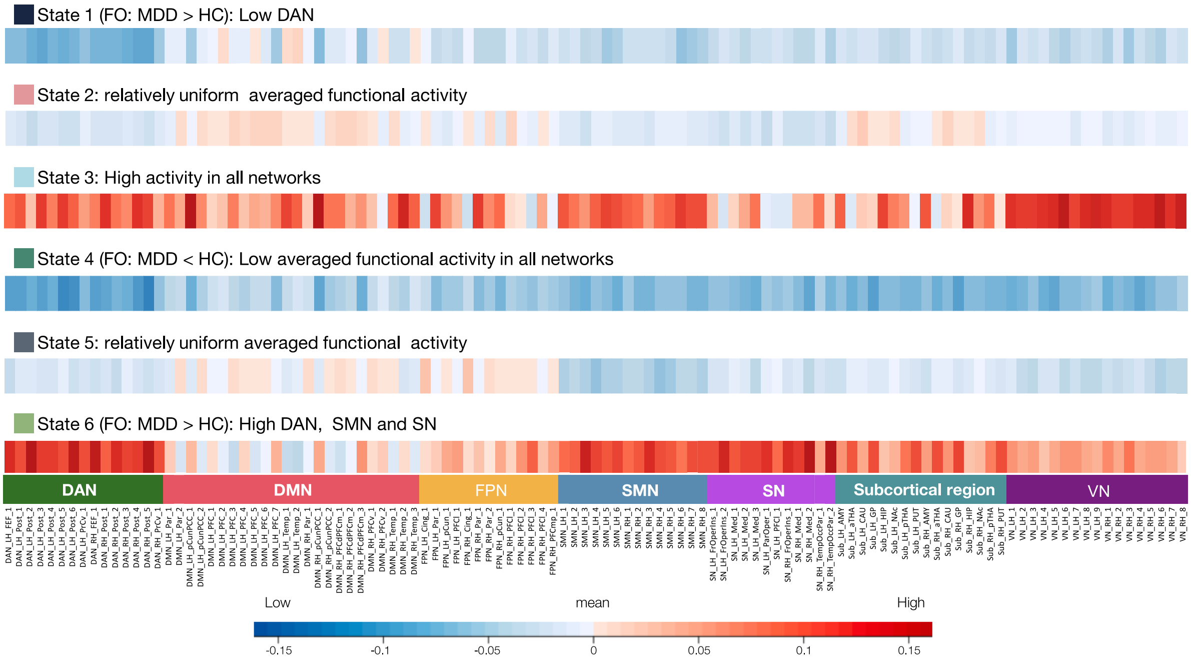


Each row of this figure represents one of the six spatial states. The columns of the heatmap are presenting the averaged functional activity of 111 brain regions, sorted by brain networks (dorsal attention network (DAN), default mode network (DMN), frontoparietal network (FPN), somatosensory motor network (SMN), Salience network (SN), Subcortical regions and visual network (VN)). Respectively, the spectrum of blue to red is indicating the negative to positive averaged functional activity. The brain maps display the averaged functional activity of the brain regions with the same value as the heatmaps (range -0.15 to 0.15).

## Supplementary Figure 4: Group comparisons of the temporal features

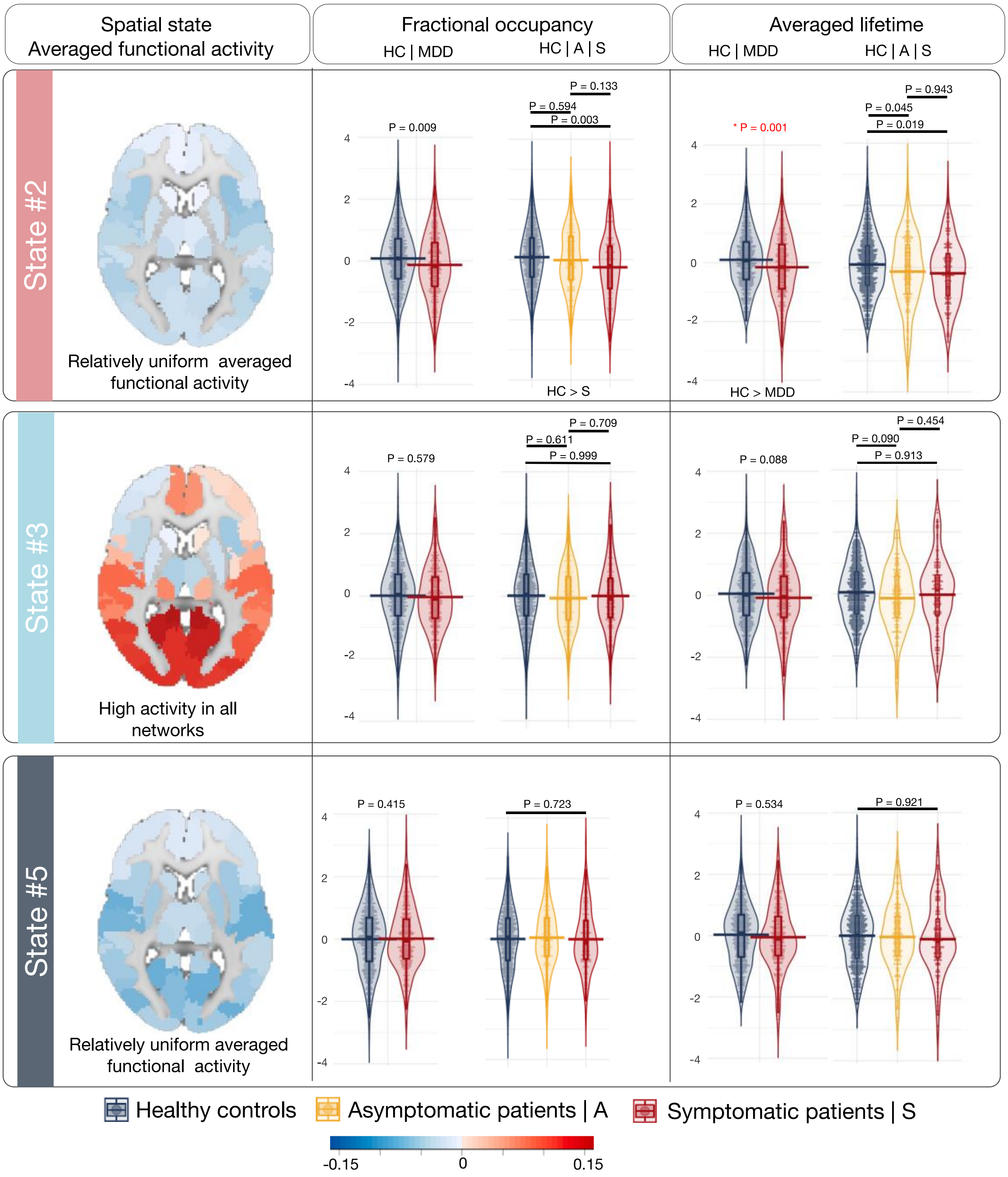


Applying the hidden Markov model (HMM) resulted in six spatial states, with the brain map of averaged functional activity represented for each state (blue to red is indicating the negative to positive averaged functional activity, range -0.15 to 0.15). This figure contains the finding of Fractional occupancy (FO) and averaged lifetime (ALT) of the

state #2, #3 and #5 and the findings related to state #1, #4 and #6 can be found in Figure 2 (main article). The range of -0.15 and 0.15 for the averaged functional activity represents the level of functional activity observed during a particular state. In general, the magnitude and direction of the values can indicate the degree and type of neural activity occurring during a particular state. The positive values may indicate increased neural activity, while negative values may indicate decreased activity. Functional activity is the averaged bold time-series at that state for each region. The violin plots represent the group comparisons (HC vs. all MDD-diagnosed patients and HC vs. asymptomatic or symptomatic patients) of the temporal features. The value on the top of each comparison is an uncorrected p-value and the p-values that are significant after the Bonferroni correction are indicated by red colour and asterisks.

**Supplementary Figure 5: Heatmap of transition probability map on the whole study population**

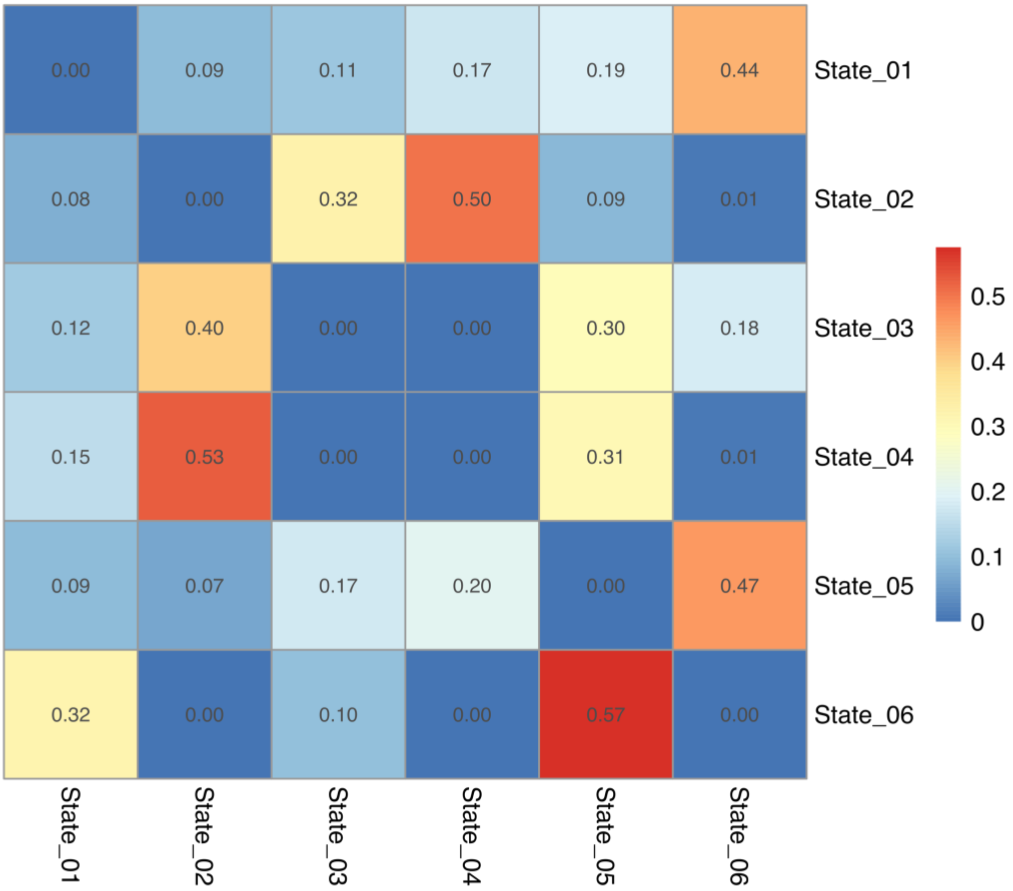


The transition probability between the states. The values show the probability value of moving from the departing state to the destination state. The spectrum of blue to red is showing the lowest value (zero: no probability) to red (over 0.5: highest probability).

**Supplementary Figure 6: Transition probability of subsets of symptomatic, asymptomatic, and healthy groups**

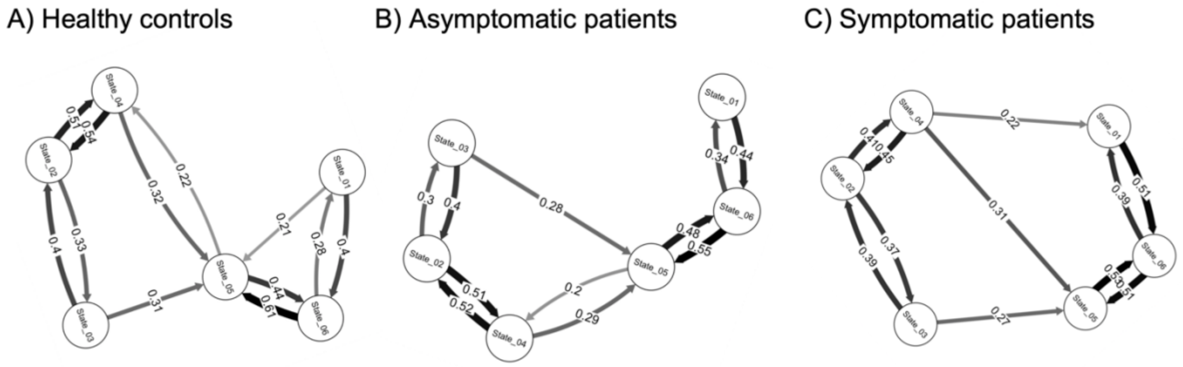


Transition probability maps. The circles are the spatial states and the directed lines with the values are the probability of moving from one state to any other state. It is possible for any participant to transition from any state to another state.

A) Healthy controls: Once the HC group moved from state #6 to state #1, they prefer to move to state #5 directly. B) asymptomatic patients have a similar transition map to healthy individuals. However, moving from state #1 to state #5 similar to the HC group is less probable for this group. C) Depression status: It is more probable for symptomatic patients to move from state #4 to state #1 directly.

**Supplementary Figure 7: Correlation of the Averaged lifetime with BDI in MDD patients**

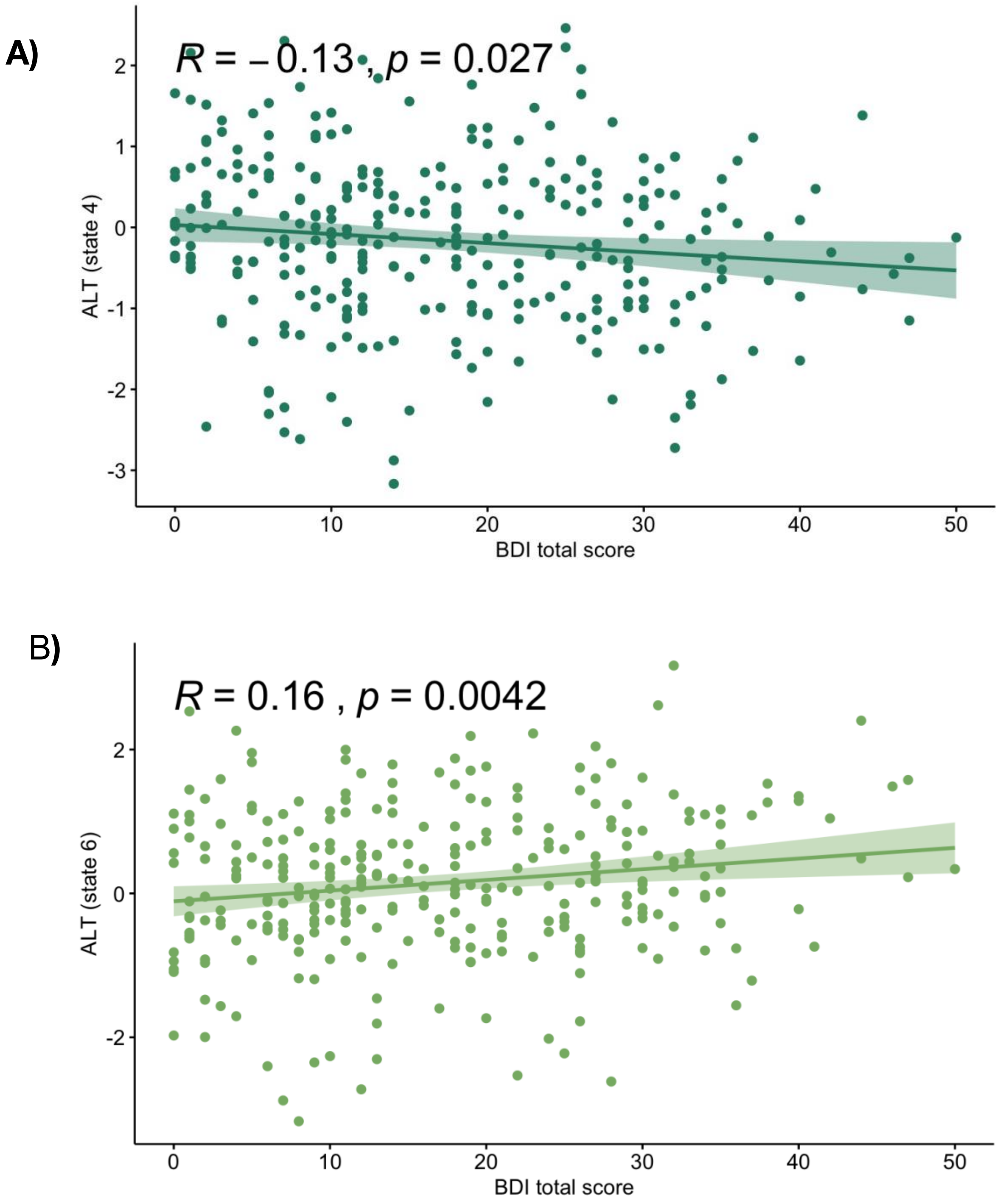


Scatter plots are showing the averaged lifetime (ALT) of states (A) #4 and (B) #6 and the BDI scores in MDD patients.

**Supplementary Figure 8: Correlation of the switching rate and total score of BDI**

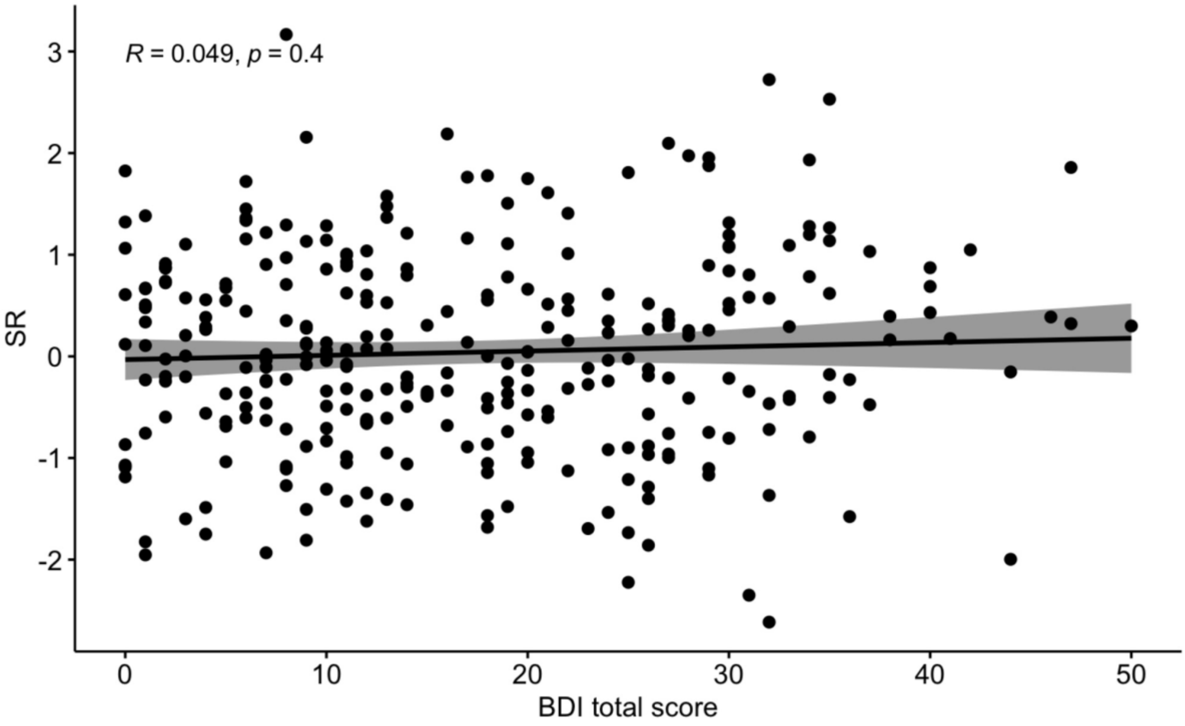


The switching rate is not significantly correlated with the total BDI score.

**Supplementary Figure 9: Pairwise correlation of the BDI factors and FO of the states**

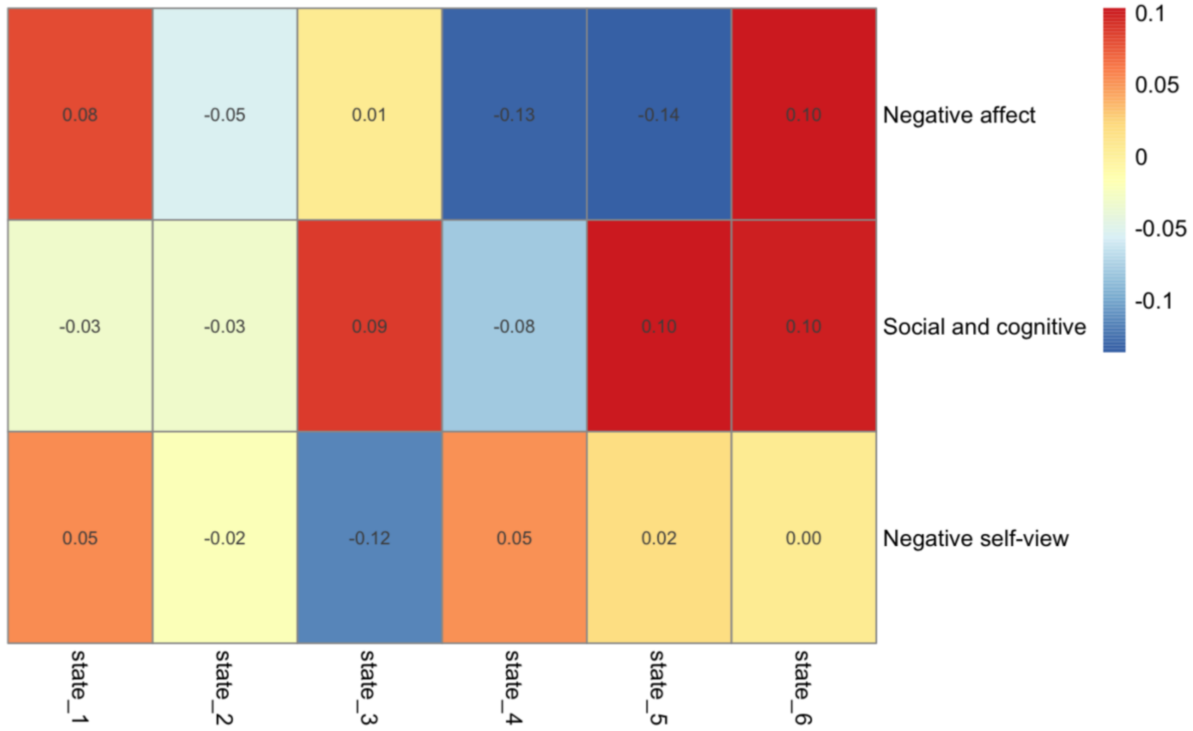


The heat map is showing the pairwise Pearson correlation (rows: eigenvalues of a BDI factor and; columns: Fractional occupancy of the states). The values printed in the heatmap are the coefficient values (r).

**Supplementary Figure 10: Pairwise correlation of the BDI factors and ALT of the states**

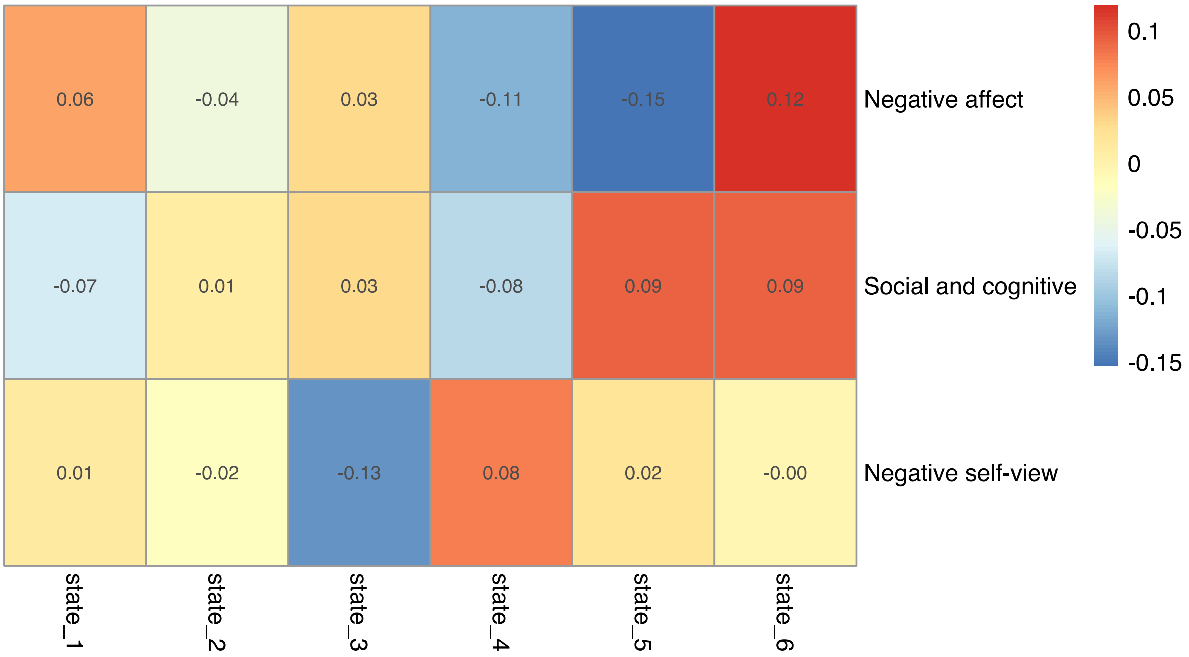


The heat map is showing the pairwise Pearson correlation (rows: eigenvalues of a BDI factor and; columns: Averaged lifetime of the states). The values printed in the heatmap are the coefficient values (r).

**Supplementary Figure 11: FO comparisons of antidepressant medicated vs. unmedicated groups in the symptomatic and asymptomatic patients**

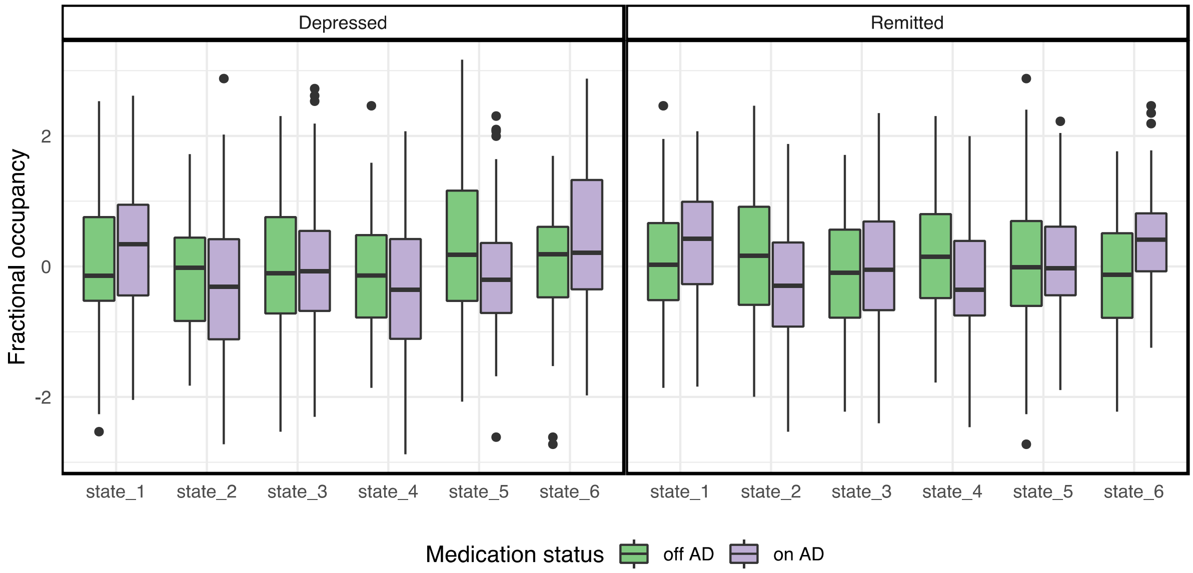


The y-axis is the percentage of fractional occupancy of each state. All the comparisons are adjusted for age and gender. The values on the top of each comparison are uncorrected p-values, and there were no significant results after the Bonferroni correction.

**Supplementary Figure 12: ALT comparisons of antidepressant medicated vs. unmedicated groups in the symptomatic and asymptomatic patients**

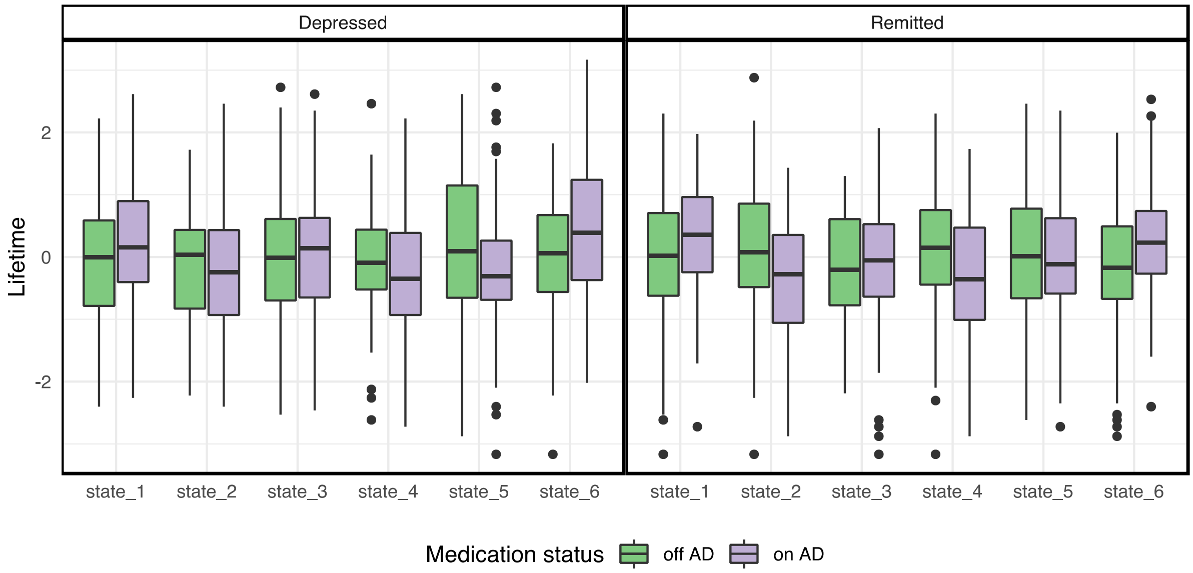


The y-axis is the percentage of Averaged life time of each state. All the comparisons are adjusted for age and gender. The values on the top of each comparison are uncorrected p-values, and there were no significant results after Bonferroni correction.

**Supplementary Figure 13: Comparisons of FO between medicated asymptomatic, and unmedicated asymptomatic, medicated symptomatic, unmedicated symptomatic, and healthy control groups**

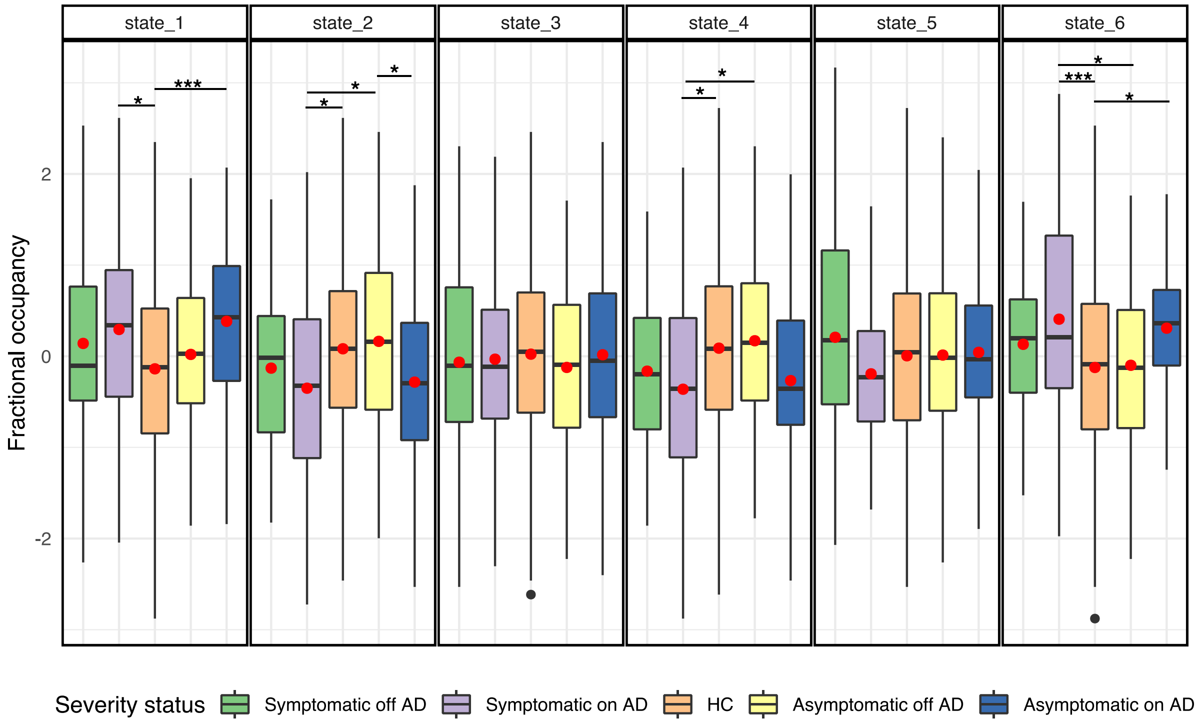


The significant p-values of pairwise comparisons adjusted for age and gender (*: $p < 0.05$, **: $p < 0.01$, ***: $p < 0.001$).

**Supplementary Figure 14: Comparisons of ALT between medicated asymptomatic, and unmedicated asymptomatic, medicated symptomatic, unmedicated symptomatic, and healthy control groups**

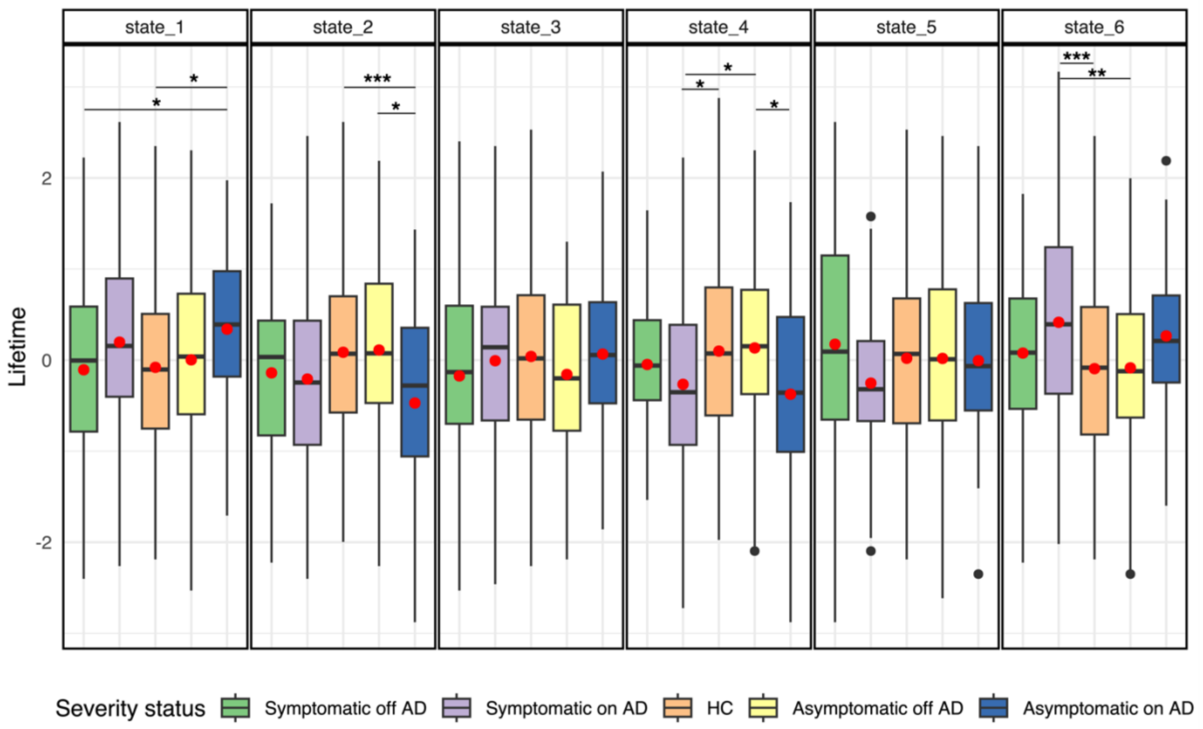


The significant p-values of pairwise comparisons adjusted for age and gender (*: $p < 0.05$, **: $p < 0.01$, ***: $p < 0.001$).
